# Qki5 safeguards spinal motor neuron function by defining the motor neuron-specific transcriptome via pre-mRNA processing

**DOI:** 10.1073/pnas.2401531121

**Published:** 2024-09-03

**Authors:** Yoshika Hayakawa-Yano, Takako Furukawa, Tsuyoshi Matsuo, Takahisa Ogasawara, Masahiro Nogami, Kazumasa Yokoyama, Masato Yugami, Munehisa Shinozaki, Chihiro Nakamoto, Kenji Sakimura, Akihide Koyama, Kazuhiro Ogi, Osamu Onodera, Hirohide Takebayashi, Hideyuki Okano, Masato Yano

**Affiliations:** ^a^Division of Neurobiology and Anatomy, Graduate School of Medical and Dental Sciences, Niigata University, Chuo-ku, Niigata 951-8510, Japan; ^b^Keio University Regenerative Medicine Research Center, Kawasaki, Kanagawa 210-0821, Japan; ^c^Department of Physiology, Keio University School of Medicine, Shinjuku-ku, Tokyo 160-8582, Japan; ^d^The Shonan Incubation Laboratory, Shonan Research Center, Takeda Pharmaceutical Company Limited, Fujisawa, Kanagawa 251-8555, Japan; ^e^Department of Animal Model Development, Brain Research Institute, Niigata University, Chuo-ku, Niigata 951-8585, Japan; ^f^Division of Legal Medicine, Department of Community Preventive Medicine, Graduate School of Medical and Dental Sciences, Niigata University, Chuo-ku, Niigata 951-8510, Japan; ^g^Department of Neurology, Brain Research Institute, Niigata University, Chuo-ku, Niigata 951-8585, Japan

**Keywords:** RNA-binding protein, Quaking5, alternative splicing, motor neuron, degeneration

## Abstract

Many RNA-binding proteins (RBPs) are linked to the dysregulation of RNA metabolism in motor neuron diseases (MNDs). However, the molecular mechanisms underlying MN vulnerability have yet to be elucidated. Here, we found that such an RBP, Quaking5 (Qki5), contributes to formation of the MN-specific transcriptome profile, termed “MN-ness,” through the posttranscriptional network and maintenance of the mature MNs. Immunohistochemical analysis and single-cell RNA sequencing (scRNA-seq) revealed that Qki5 is predominantly expressed in MNs, but not in other neuronal populations of the spinal cord. Furthermore, comprehensive RNA sequencing (RNA-seq) analyses revealed that Qki5-dependent RNA regulation plays a pivotal role in generating the MN-specific transcriptome through pre-messenger ribonucleic acid (mRNA) splicing for the synapse-related molecules and c-Jun N-terminal kinase/stress-activated protein kinase (JNK/SAPK) signaling pathways. Indeed, MN-specific ablation of the Qki5 caused neurodegeneration in postnatal mice and loss of Qki5 function resulted in the aberrant activation of stress-responsive JNK/SAPK pathway both in vitro and in vivo. These data suggested that Qki5 plays a crucial biological role in RNA regulation and safeguarding of MNs and might be associated with pathogenesis of MNDs.

## Significance

RNA-binding proteins (RBPs) play pivotal roles in a cellular RNA metabolism by regulating multiple steps of the posttranscriptional gene regulation and its functional abnormality is linked to pathogenesis of various diseases. Most of RBPs are widely expressed across tissues and this would suggest a role of supporting a basic cellular function. On the other hand, the molecular mechanisms underlying cell type-specific transcriptome and molecular features related to the selective cell vulnerability remain to be elucidated. Here, we describe a role of Quaking5 (Qki5) in the generation of the motor neuron (MN)-specific transcriptome profile through pre-messenger ribonucleic acid (mRNA) regulation. Moreover, Qki5 contributes to the maintenance of MNs, and its dysfunction is associated with MN vulnerability.

Motor neurons (MNs) are specialized neurons that regulate the movement mediating synaptic connections with muscles. Various MN diseases, such as spinal muscular atrophy and amyotrophic lateral sclerosis (ALS), are thought to be caused in part by dysfunction of RNA-binding proteins (RBPs) including fused in sarcoma (FUS) and TAR DNA–binding protein 43kDa (TDP-43). These RBPs are often ubiquitously expressed in various tissues and cell types, reflecting the previous report that the majority of ~1,524 genes encode RBPs in humans are ubiquitously expressed across tissues relative to DNA-binding proteins (1). RBPs that are specifically expressed in neurons, such as neuro-oncological ventral antigen (Nova) and nElavls, are known to regulate the transcriptome related to neuronal function and neurogenesis (2, 3), but they are not specialized RBPs that generate the MN-specific transcriptome. The Quaking (Qki) protein is a member of the STAR family of KH domain-containing RBP that shows cell-type specific expression. Their splicing variants of Qki proteins (Qki5, 6, and 7) are widely expressed across several tissues from embryos to adults (4–6). In the nervous system, Qki proteins are expressed in glial cells (7, 8) and are key regulators of the maturation of oligodendrocytes (OLs) and astrocytes. This regulation is mediated by posttranscriptional regulation of cell cycle- and myelin-related genes and astrocyte-related genes (9–14). We and another group have recently revealed that Qki5 is expressed in neural stem cells (NSCs) (13, 15) Furthermore, we showed that Qki5 is an essential factor for the maintenance of early embryonic NSCs through pre-messenger ribonucleic acid (mRNA) regulation in tight junctions and cell adhesion pathways (15).

In the present study, we report Qki5 protein expression in spinal MNs and biological roles of Qki5 in MNs. Detailed immunostaining using the developing mouse spinal cord and single-cell RNA (scRNA)-seq analysis using human-induced pluripotent stem cell-derived MNs (hiPSC-MNs) revealed that Qki5 is predominantly expressed in MNs of neuronal population in the spinal cord. Transcriptome analysis with loss of Qki5 function suggested that Qki5 contributes to the generation of the MN-specific transcriptome, hereafter referred to as MN-ness. Furthermore, we confirmed that Qki5 deficiency induced alternative splicing dysregulation of the cytoskeleton- and synapse-related molecules via gene ontology pathway analysis using a list of direct targets of Qki5. By mouse genetics and cell biological assays, we found that MN-specific deletion of Qki5 resulted in MN degeneration in vivo and cultured MNs with *Qk* knock-down caused an aberrant activation of the stress-responsive c-Jun N-terminal kinase/stress-activated protein kinase (JNK/SAPK) pathway. Collectively, Qki5 is essential for generation of the MN-specific transcriptome profile and maintenance of matured MNs through posttranscriptional regulation.

## Results

### Qki5 Protein Is Expressed in Postmitotic Spinal MNs to Adult Stage.

**Fig. 1.**
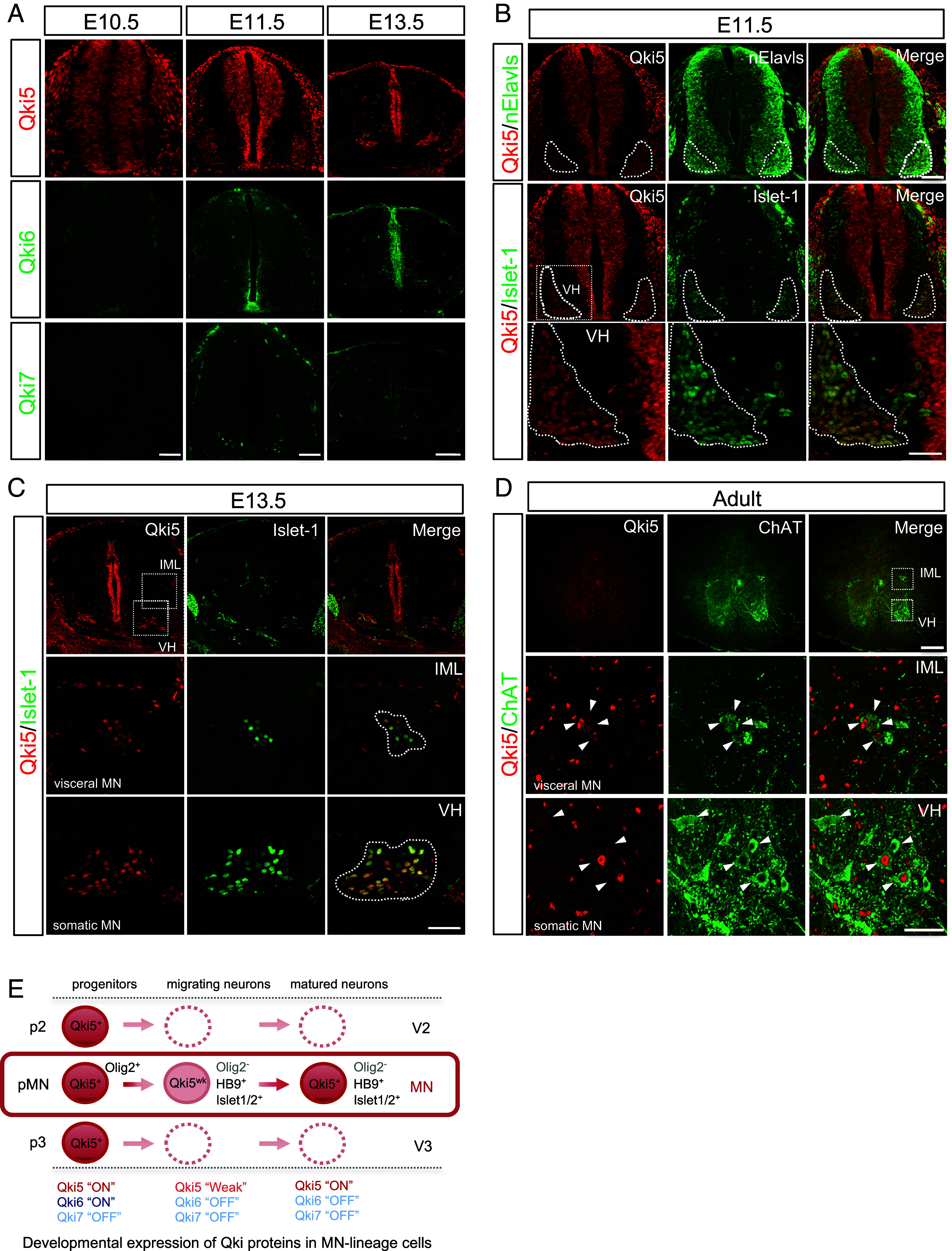
Qki5 protein is expressed in MNs in the mouse spinal cord. (*A*) Transverse sections of mouse spinal cords at each developmental age embryonic day (E) 10.5, E11.5, and E13.5 were immunostained using isoform-specific antibodies (Qki5, Qki6, and Qki7), showing a specific expression pattern of Qki5 among proteins encoded by the *Qk* gene. (Scale bar, 50 μm for E10.5, 100 μm for E11.5, and 150 μm for E13.5.) (*B*) Detailed expression of Qki5 and lineage marker proteins of MNs in the E11.5 mouse spinal cord. Qki5 (red) emerged in nElavls-positive (green, *Top*) and Islet-1-positive (green, *Middle*, and *Bottom*) MN pools. The *Bottom* panels represent the enlarged views of the ventral horn (VN) indicated by *Insets* in the *Middle-Left* panel. Note that Qki5 is expressed in MNs of the ventral horn, but not in other types of neurons. (Scale bar, 100 μm for *Top* and *Middle* and 50 μm for *Bottom*.) (*C*) Qki5 (red) is expressed in both somatic MNs of the ventral horn (VH) and visceral MNs of the intermediolateral nucleus (IML) labeled with Islet-1 (green) in the E13.5 mouse spinal cord. The *Middle* and *Bottom* panels represent the enlarged view of the IML and ventral horn (VN) indicated by *Insets* in the *Top-Left* panel. (Scale bar, 150 μm for the *Top* and 50 μm for the *Middle* and *Bottom*.) (*D*) Transverse sections of the adult mouse spinal cord were immunostained using anti-Qki5 (red) and ChAT (green) antibodies. The *Middle* and *Bottom* panels represent the enlarged views of the IML and ventral horn (VH), respectively, indicated by the *Insets* in the *Top* panel. (Scale bar, 200 μm for the *Left* and 50 μm for the *Top* and *Middle*/*Bottom* panels.) (*E*) Schematic illustrations of the expression patterns of Qki proteins in MN-lineage cells. Qki5 is expressed in embryonic NSCs of progenitor domains, including the pMN domain labeled with Olig2. After that, its expression is weakened in migrating Olig2-negative, Islet-1, and HB9-positive MNs and restored in both somatic and visceral MNs, which are settled in the final destination. In contrast, the Qki6 and Qki7 proteins were not detected in any neuronal population.

Qki RBPs regulate the differentiation of glial cells, such as OLs and astrocytes in the central nervous system (CNS) (6, 10). We recently reported that Qki5 is transiently expressed in early embryonic NSCs and involved in cell adhesion signaling through pre-mRNA splicing (15), suggesting that cell type-specific Qki5 expression enables lineage-committed cells to drive the cell type-specific transcriptome and the acquisition of cell type-specific functions. Therefore, we assessed the expression of Qki proteins in the developing mouse spinal cord in detail by immunofluorescence (IF) using previously validated isoform-specific antibodies (15). We observed that Qki5 is likely to be expressed in the neuronal population of the ventral horn in addition to NSCs in the mouse spinal cord at embryonic day 11.5 (E11.5), whereas Qki6 and Qki7, other alternative splicing isoforms, were not detected in this area (Fig. 1*A*). Detailed IF analyses revealed unique expression of Qki5 protein. Although Qki5 protein was undetectable in the nElavls-positive neuronal population, including MNs marked by Islet1 or HB9 (Mnx1) at E10.5 (*SI Appendix*, Fig. S1*A*), Qki5 expression emerged in migrating MNs and MNs settled in the ventral horns at E11.5 (Fig. 1*B*). Interestingly, Qki5 expression was specific to MNs and not observed in other nElavls-positive neurons including dl3 interneurons (another Islet1-positive population) (Fig. 1*B*). We further confirmed that both visceral MNs and somatic MNs expressed the Qki5 protein in the E13.5 mouse spinal cord (Fig. 1*C* and *SI Appendix*, Fig. S1*B*) and the expression is maintained in Choline acetyltransferase (ChAT)-positive MNs to adult stage (Fig. 1 *D* and *E* and *SI Appendix*, Fig. S2*A*). In motor subtypes, Qki5 expression was observed in both ChAT^+^/Rbfox3^+^ α-MNs and ChAT^+^/Rbfox3^-/weak^ γ-MNs (*SI Appendix*, Fig. S2*A*) (16, 17). Importantly, Qki5 expression was not detected in GAD67- or Rbfox3 (NeuN)-positive dorsal interneurons, while Qki5 is largely coexpressed with OL lineage protein Olig2 (*SI Appendix*, Fig. S2 *B*–*D*).

**Fig. 2.**
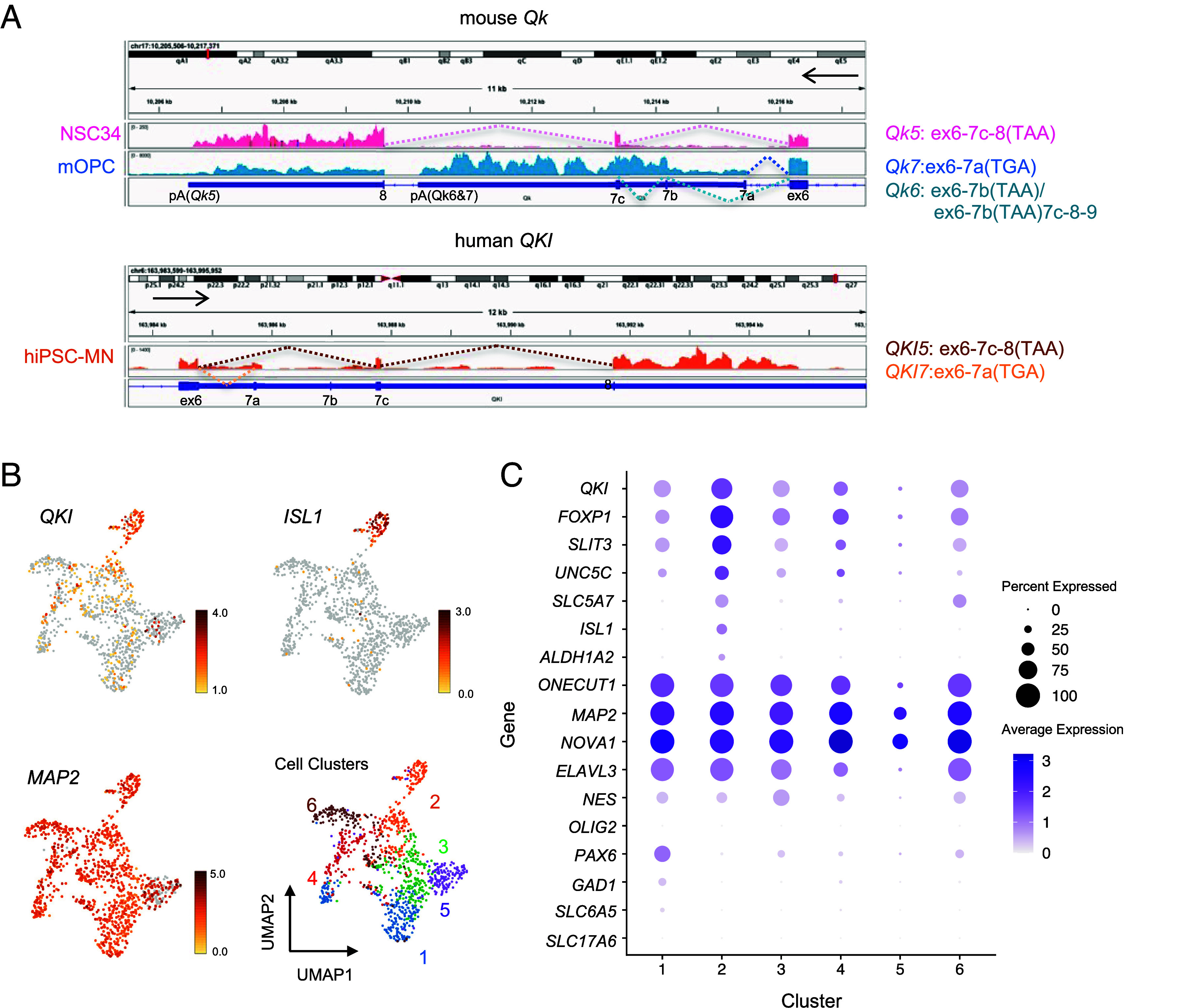
*Qki5* expression in mouse and human spinal MNs. (*A*) RNA-seq analyses were performed using mouse-MN-like NSC-34 cells, mouse OL precursor cells (mOPCs), and hiPSC-MNs. Integrative Genomics Viewer (IGV) image of alternatively spliced *Qk* transcripts (5, 6, and 7) in NSC-34 cells, mOPCs, and hiPSC-MNs. mMNs and hiPSC-MNs predominantly express *Qk5* and *QKI5*, respectively. In contrast, mOPCs equally express all three *Qk* isoforms. The alternative splicing pattern of each isoform is shown on the *Right*. (*B*) UMAP of scRNA-seq using hiPSC-MNs showing six different clusters. *QKI* and *ISL1* are dominantly expressed in cluster 2, whereas *MAP2* is expressed in all clusters. (*C*) Seurat’s dot plot showing expression of *QKI* and 16 marker genes in the scRNA-seq. Dot color intensity and dot size represent average expression of the gene and the percentage of cells expressing the gene, respectively in a given cluster.

To further verify predominant Qki5 expression in MNs, we performed RNA sequencing (RNA-seq) analysis using NSC-34 mouse MN-like (mMN) cells, mouse primary cultured OPCs (mOPCs), hiPSC-MNs, and additional public RNA-seq datasets (18–21). *Qk* mRNA is predominantly expressed in compared to *Elavl3*, encoding panneuronal RBP (*SI Appendix*, Fig. S2*E*). In addition, Mouse and human MNs predominantly expressed *Qk5* and *QKI5*, respectively. On the other hand, mOPCs and floorplate expressed all isoforms (Fig. 2*A* and *SI Appendix*, Fig. S2*F*). For a deeper analysis of MN expression of *QKI5*, we performed a single-cell transcriptome analysis using hiPSC-MNs model that we previously published (22). Dimensionality reduction by Uniform Manifold Approximation and Projection (UMAP) revealed that our hiPSC-MNs model could be divided into six different cell clusters (Fig. 2*B*). Almost the cell population was committed to neurons based on the expression of *ELAVL3/MAP2/NOVA1* except cluster 5. In addition, these cell clusters except cluster 5 were negative for *OLIG2* which expresses in motor progenitor (pMN) and positive for *ONECUT1* which are expressed from immature MN to mature MNs, suggesting the possibility that this model could be a population committed to MNs after final cell division. Further, *QKI* mRNA-expressing cells also showed an overlap with cells expressing high levels of *ISL1*, one of the best-established markers of MNs and a similar expression with ISL1 downstream genes, such as *FOXP1* and *ALDH1A*, as well as MN genes, *SLIT3*, *UNC5C,* and *SLC5A7* (Fig. 2 *B* and *C*). However, neither glutamatergic neuron markers, such as *VGLUT1* and *VGLUT2*, nor interneuron markers, such as *En1* (V1), *CHX10* (V2), and *SIM1* (V3), were not observed in most cell clusters except for cluster 1. On the other hand, cluster 1 included *PAX6*^+^/*LHX*^+^/*PAX2*^+^ V0_D_ neurons and *PAX6*^+^/*LHX*^+^/*PAX2*^+^ V0_V_ neurons, which are expressing gamma-aminobutyric acid-expressing (GABAergic) neuron marker, *GAD1*, and/or glycinergic neuron marker, *SLC6A5* (Fig. 2 *B* and *C* and *SI Appendix*, Fig. S2*G*).

### Qki5 Contributed to the Generation of the MN-Specific Transcriptome.

To investigate how Qki5 functions in MNs, we performed transcriptome analysis of mRNA obtained from mMNs, hiPSC-MNs, and mOPCs, as a nonneuronal lineage cell control, which expresses all Qki proteins (Fig. 2*A*). mMNs, hiPSC-MNs, and mOPCs were transfected with three different siRNAs (*siQk* #1 to 3) or control siRNAs to knock down (KD) the *Qk* or *QKI* transcripts in cultures. Seventy-two hours after transfection, the cells were collected and subjected to NGS analysis. We generated mRNA libraries from three independent NSC-34 cultures and performed 75 bp paired-end sequencing on the Illumina Nextseq 500 system. In total, 147 and 151 million paired-end reads were obtained for the control and *Qk* siRNA mRNA-seq samples, respectively. Sequence reads were aligned to the mouse genome (mm10) and human genome (hg19) or an exon-junction database with the OLego tool, and gene expression levels and alternative splicing events were quantified with the Quantas tool (23). We confirmed that the *Qk* or *QKI* transcripts were efficiently down-regulated in each cell (*SI Appendix*, Fig. S3).

**Fig. 3.**
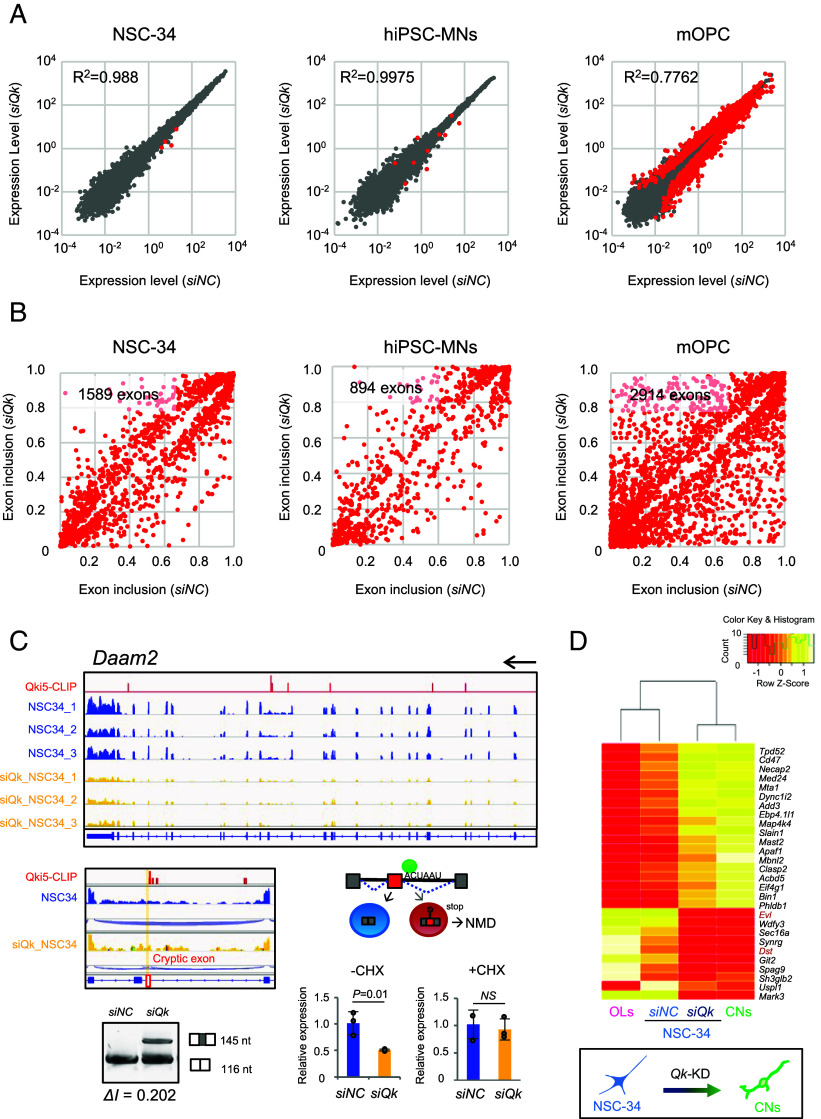
Qki5 regulates pre-mRNA splicing of a subset of transcripts in mouse and human MNs. (*A*) Scatterplot of the Log10 RPKM of *siNC* vs. *siQk* RNA-seq (expression levels: logCPM > 0) in NSC-34 cells, hiPSC-MNs, and mOPCs. Each dot in the plot indicates an individual transcript, and significant hits for transcript level change (*P* < 0.01 and FDR < 0.1) are indicated by red color. Only four transcripts and 87 transcripts were changed in Qki5-expressing NSC-34 cells and hiPSC-MNs, respectively, relative to the dramatic change in mOPCs. Each point represents the mean RPKM value obtained from three biological replicates for an individual gene. Each R-squared value is denoted in the graphs. (*B*) Scatterplot of the exon inclusion of *siNC* vs. *siQk* RNA-seq significant hits (*P* < 0.01 and FDR < 0.01) with absolute fold changes of 1.5 or more. Each point represents the ratio of read counts obtained from three replicates for an individual alternative splicing event. The number of changed alternative splicing events is denoted in each graph. (*C*) Representative IGV view of alternative splicing changes in the *Daam2* gene in *siNC* and *siQk* NSC-34 cells with Qki5 HITS-CLIP cluster. *siQk* cells show that a cryptic exon induces nonsense-mediated decay (NMD). Gel images for RT–PCR validation of alternative splicing between exon 15 and 16. Bar graphs indicate the qRT-PCR assay of the relative expression of *Daam2* transcript adjusted to the internal control *Gapdh* using *siNC* and *siQk* NSC-34 cells with or without cycloheximide (CHX) treatment. Data represent the mean ± SD from three independent biological replicates, two-tailed Student’s *t* test. (*D*) A dendrogram and heat-map overview of the hierarchical cluster analysis of alternative exon usage in genes from mOPC-, *siNC*-, or *siQk*-transfected NSC-34 cells and mouse cortical neurons (CNs) (n = 3 for each) using 28 Qki5-regulated OPC-specific exons (*Top*). Columns represent individual samples, and rows represent each exon. Each cell in the matrix represents the expression level of an alternative exon in an individual sample. The color key and histogram indicate that red and yellow in cells reflect high and low expression levels, respectively.

First, we analyzed the mRNA-seq data at the transcript level. There were few changes in the degree of gene expression between si-negative control (*siNC*) and *siQk* KD in both NSC-34 cells (R^2^ = 0.988) and hiPSC-MNs (R^2^ = 0.9975) (Fig. 3*A*). We observed steady-state level changes in only 3 of 23,038 transcripts that were down-regulated (cutoff: logCPM > 0, *P* < 0.01, and FDR *P* < 0.1) in NSC-34 cells. In addition to the *Qk* gene (logFC = 2.96), *L3hypdh* (*2810055F11Rik*, logFC = 1.74), *Daam2* (logFC = 1.40), and *Snap23* (logFC = 1.20) were down-regulated at the transcript level in *siQk* KD NSC-34 cells (Fig. 3*A* and *SI Appendix*, Table S1). Additionally, the levels of 87 of 14,528 total transcripts were changed in *siQKI* hiPSC-MNs (cutoff: logCPM > 0, *P* < 0.01, and FDR *P* < 0.1), similar to NSC-34 cells (Fig. 3*A* and *SI Appendix*, Table S2). In contrast, mOPCs subjected to *Qk*-KD showed many transcript level changes (R^2^ = 0.7762). This could be due to the effect of not only Qki5 but also Qki6 and 7, which contribute to cytoplasmic functions, such as mRNA stabilization and translation in OLs.

To further determine whether *Qk*-KD affects pre-mRNA processing events in these cells, we analyzed mRNA-seq data at the exon-junction level. As expected, we identified dramatic mRNA processing defects, which could be due to changes in potential Qki5-dependent mRNA processing events in NSC-34 cells (1,589 exons), hiPSC-MNs (894 exons), and mOPCs (2,914 exons) (Fig. 3*B*). These differences included all types of alternative exon changes in NSC-34 cells and hiPSC-MNs (i.e., cassette exons, mutually exclusive exons, tandem cassette exons, alternative 5′ sites, alternative 3′ sites, and intron retention) (*SI Appendix*, Tables S3 and S4). *Qk* KD primarily affected cassette-type exons (64.0% and 57.1% in NSC-34 cells and hiPSC-MNs, respectively) with a similar tendency in embryonic NSCs (15). We next investigated the Qki5-dependent nonannotated exon inclusion by focusing on the genes with transcript level change, *L3hypdh, Daam2*, and *Rab12* (Fig. 3*C* and *SI Appendix*, Fig. S4 *A* and *B*), like the loss of TDP-43 function causing a cryptic exon inclusion (24, 25). Interestingly, we found that the *Daam2* gene contained a bona fide cryptic exon between exons 15 and 16 in *siQk* NSC-34 cells, which included a premature stop codon, thereby causing mRNA degradation via the nonsense-mediated decay (NMD) pathway. Furthermore, we observed the inclusion of nonannotated exons in a 1st exon extension, and a retained intron between exon 3 and exon 4 in the *L3hypdh* gene and between exons 2 and 3 of *Rab12* in *siQk* NSC-34 cells (*SI Appendix*, Fig. S4 *A* and *B*). All these exons surrounded ACUAAY codes, as evidenced by HITS-CLIP analysis, suggesting that Qki5 fine-tuned its transcript level through this alternative exon regulation (Fig. 3*A* and *SI Appendix*, Fig. S4 *A* and *B*). Importantly, we confirmed by qRT–PCR assay that cycloheximide (CHX) treatment, which can inhibit the NMD pathway, restored the down-regulated transcript levels in *Qk*-KD cells. Therefore, we concluded that these transcript level changes resulted from Qki5-dependent AS changes in cells, again indicating that the primary function of nucleus-localized Qki5 is pre-mRNA splicing.

The above data from histological and transcriptomic analyses suggested that Qki5 is a cell-type-specific RBP that regulates alternative splicing to define the MN-specific transcriptome in MNs but not in other neurons. On the other hand, Qki5 is also a well-known RBP that regulates OL differentiation through mRNA regulation. To determine the extent to which Qki5 contributes to cell-type-specific alternative splicing in OLs and MNs, we first extracted 164 OPC/OL-specific alternative splicing events compared to cortical neurons (CNs) using public transcriptome datasets of glial cells, neurons, and vascular cells of the cerebral cortex (18). Among those cells, we assessed the Qki5 contribution of OPC-specific exons and found that notably, Qki5 regulates approximately 51.8% of OL-specific exons (79 Qki5-regulated exons of 164 OPC-specific exons) in mOPCs (*SI Appendix*, Fig. S4*C*). Next, we compared the expression levels of these 28 out of 79 Qki5-regulated OPC-specific exons that were selected by a criterion of *ΔI* > 0.2 changed exons between NSC-34 and *siQk*-NSC34 and performed hierarchical cluster analysis of alternative exon usage in OLs, *siNC* (ctrl), or *siQk*-KD NSC-34 cells, and mouse CNs (n = 3 for each). Interestingly, NSC-34 MN-like cells and OLs showed more robust clustering than CNs in terms of OLs-specific alternative exon usage (Fig. 3*D*). To further explore the Qki5 dependency of OLs-specific exon usage in MNs, we compared alternative splicing between control and *Qk*-KD NSC-34 cells. As a result, *Qk*-KD NSC-34 cells showed a similar CNs-like alternative splicing usage pattern to control NSC-34 cells. Indeed, the exon usage of two representative *Dst* and *Evl* genes is correlated with the predominant expression of Qki5 in MNs rather than those in CNs and several types of embryonic stem cell (ESC)-derived interneurons, which do not express Qki5 protein (*SI Appendix*, Fig. S4*D*). These data indicate that Qki5 can regulate MN- and OPC-OL-specific transcriptomes and that these two morphologically and functionally distinct cell types could share a common alternative splicing usage. Indeed, this would make sense since MNs and OPCs share a cell lineage: they are derived from a common Olig2-positive progenitor in the pMN domain of the ventral part of the spinal cord.

### Transcriptome Analysis Reveals Biological Pathways Regulated by Qki5-Dependent Alternative Splicing in MNs.

Our finding that Qki5 contributes to the MN-specific transcriptome through mRNA processing raises the question of whether Qki5-dependent RNA targets could predict the biological significance of Qki5 in MNs. To further elucidate the biologically relevant pathway of the Qki5 RNA targets, we first performed Metascape pathway analysis using a list of genes that have alternative splicing changes between *siNC* and *siQk* KD in mMNs (660 changed AS exons in *siNC* vs. *siQk*, cutoff: *P* < 0.01, FDR < 0.1, |DI| > 0.05, and reads per kilobase of exon per million mapped reads (RPKM) > 1) and hMNs (388 changed AS exons in *siNC* vs. *siQKI*, cutoff: *P* < 0.01, FDR < 0.01, |DI| > 0.05, and RPKM > 1). The following pathways were also ranked among the top five enriched gene ontology (GO) terms and pathways in NSC-34 cells: #1: mRNA processing (LogP −14.7), #2: organelle localization (LogP −13.4), #3: regulation of cell projection organization (LogP −10.9), #4: positive regulation of organelle organization (LogP −10.5), and #5: splicing factor NOVA-regulated synaptic proteins (LogP −9.6) (*SI Appendix*, Fig. S5*A* and Table S5) (15). In hiPSC-MNs, the following transcripts were ranked among the top five: #1: actin filament-based process (LogP −23.2), #2: cellular component morphogenesis (LogP −20.6), #3: cell junction organization (LogP −20.3), #4: signaling by Rho GTPase, Miro GTPase, and RHOBTB3 (LogP −13.7), and #5: Nervous system development (LogP −9.6) (*SI Appendix*, Fig. S5*B* and Table S6). As mentioned above, we observed commonly enriched GO terms between NSC-34 cells and hiPSC-MNs. Moreover, in the illustration of enriched GO clusters, these top-ranked GO terms and pathways were linked to each other (*SI Appendix*, Fig. S5). Interestingly, Qki5-regulated AS targets were related to panneuronal “splicing factor NOVA-regulated synaptic proteins” in NSC-34 and hiPSC-MN cells. Moreover, Qki5-regulated exons were significantly enriched in synapse-related GO terms and pathways, e.g., neurexins and neuroligins, dendrite morphogenesis, the splicing factor NOVA regulates synaptic proteins, and chemical synaptic transmission, especially in hiPSC-MNs. This suggests that Qki5 contributes alternative exons related to synapse formation and synaptic transmission in MNs.

Next, we focused on synapse-related genes and MN disease (MND)-linked genes to understand Qki5 function in MNs (*SI Appendix*, Fig. S6). The *Nrxn* and *CASK* genes among the Neurexin1 and Neuroligin pathways contain several Qki5–RNA-binding sites were identified by Qki5 HITS-CLIP. One large Qki5–RNA-binding cluster containing the ACUAAC site was identified in the *Nrxn1* gene, which was located in intronic region upstream of alternative exons (called SS2A exon 7) inhibited by Qki5 (*SI Appendix*, Fig. S6*A*). This exon encodes 8 amino acids with a β-sheet structure in the laminin G-like 2 domain, which stabilizes the interaction with Neurexophilin (Nxph1) (26). In the case of the *CASK* gene, we also observed Qki5–RNA-binding clusters containing the ACUAAU site upstream of alternative exon 19 (*SI Appendix*, Fig. S6*B*). This exon 19 is located between the PDZ domain and SH3 domain, which are essential for the neurexin interaction (27, 28), and Qki5 is involved in this exon 19 exclusion. Therefore, these Qki5-dependent AS events might be linked to neurexin–neuroligin transsynaptic cell adhesion and synapse formation. Interestingly, this Qki5-dependent regulation in mouse MN-like cells was well conserved in hiPSC-MNs, as validated by a semiquantitative RT–PCR assay (*SI Appendix*, Fig. S6 *A* and *B*). In addition, we focused on the ALS-related genes *NEK1* (29, 30), *TAK1* (31), and *AGRN* (Y-exon, encoding the sequence lysine, serine, arginine, lysine: Y site), which are necessary for agrin–heparin interactions (32). Neural isoforms containing a heparin-binding site (Y+) and all muscle-derived isoforms are dispensable for major steps in synaptogenesis (*SI Appendix*, Fig. S6*C*) (33). These alternative splicing events were validated by a gain-of-function study using the DOX-inducible QKI5-expressing human HEK293-FRT cell line (*SI Appendix*, Fig. S6*C*).

### *Qk* KD Induced an Aberrant JNK Pathway Activation.

**Fig. 4.**
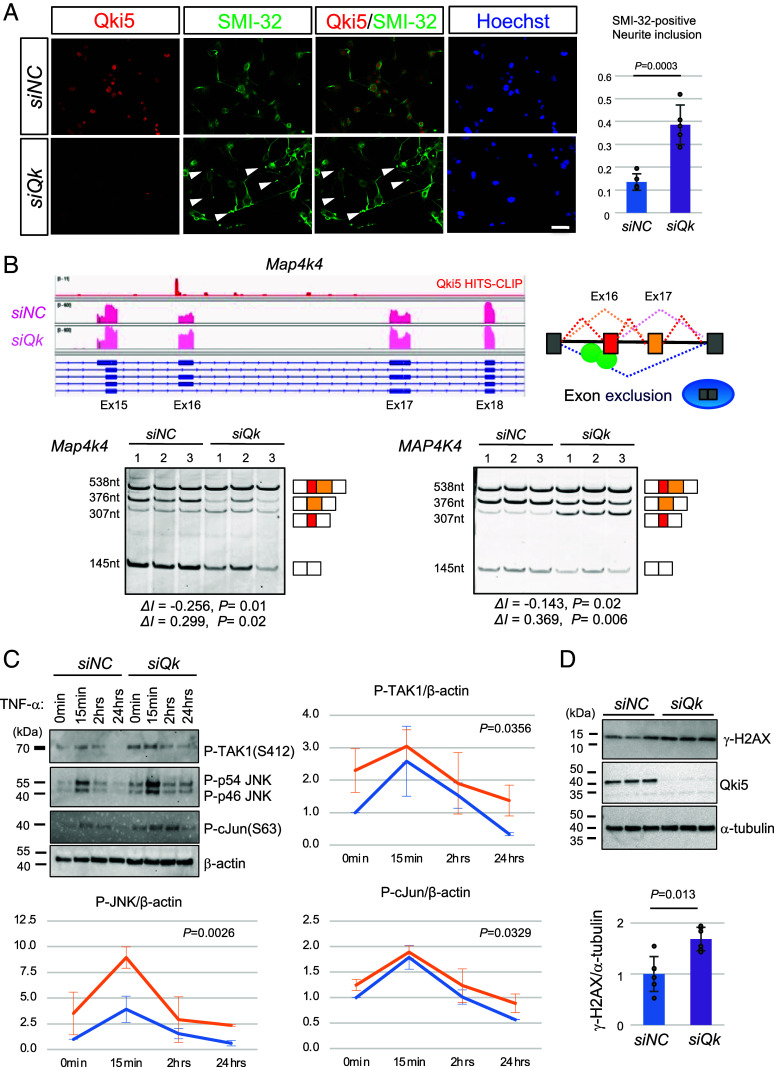
Aberrant activation of JNK/SAPK signaling in MNs with *Qk* knockdown. (*A*) Double immunostaining of Qki5 (red) and SMI-32 (green) in differentiated NSC-34 cells transfected with *siQk* or *siNC*. Forty-eight hours after siRNA transfection, Qki5 protein expression was markedly decreased in *siQk* cells, and SMI-32-positive inclusions were evident in the neurites of the cells (indicated by arrowheads) (*Left*). (Scale bar, 50 μm.) Bar graph showing that significant increase of SMI-32-positive inclusions in *Qk* KD differentiated NSC-34. Data represent the mean of five independent experiments (total 189 cells and 168 cells counted for *siNC* and *siQk*, respectively) Each dots represent average number of SMI-32 positive inclusion adjusted for the cell number in each replicate. Student’s *t* test. (*B*) Qki5-dependent AS regulation in mouse *Map4k4* and human *MAP4K4*. Schematic representation of Qki5 binding position-dependent alternative exon usage (tandem cassette exons, exon 16 and exon 17) in the mouse transcripts in the *Top-Right* panel. IGV image of Qki5 HITS CLIP clusters and expression levels of the transcript in *siNC* and *siQk* NSC-34 cells in the *Top Left* panel. Qki5 HITS-CLIP clusters were found upstream and on alternative exon 16 of the transcripts and inhibited exon 16 usage. RT–PCR validation assays were performed to monitor the effect of Qki5 on *Map4k4* (*Bottom Left*). Similar regulation was observed for human *MAP4K4*. Qki5-dependent exon exclusion was confirmed by three independent biological replicates. (*C*) Time course assay for activation of JNK/SAPK signaling with TNF-α stimulation in control and *Qk*-KD cells. Western blot indicated elevated JNK/SAPK signal transduction in *Qk*-KD cells under basal and TNF-α-treated conditions. *Qk*-KD causes increased phosphorylation of TAK1 (S412), JNKs (T183/Y185), and c-Jun (S63). Line graphs showing significant upregulation of phosphorylated (P)-TAK1, JNK, c-Jun normalized with β-actin. Data represent mean ± SD. Two-way ANOVA. (*D*) Western blot analysis of γH2AX expression in *siNC* and *siQk* cells. Note that *Qk* deletion induced γH2AX expression (*Top*). The bar graph shows the quantification of the γH2AX protein relative to α-tubulin (*Bottom*). Values were normalized to α-tubulin to obtain relative densitometric intensity. Data represent the mean ± SD of three independent experiments, two-tailed Student’s *t* test.

To explore the causative pathway of the loss of Qki5 function, we used a cell culture model, differentiated NSC-34 MN-like cells with *Qk* knockdown in vitro. We confirmed the 89.6% downregulation of endogenous Qki5 protein in NSC-34 cells by Western blots (*SI Appendix*, Fig. S7 *A*–*C*). NSC-34 cells were differentiated by retinoic acid and transfected with *siQk* or *siNC*, and the cells were subjected to immunocytochemistry and assays for protein properties 2 d after transfection. The IF study revealed that *Qk* KD cells displayed SMI-32-positive spheroids and cell death labeled with SYTOX green and active Caspase 6 signal in neurites, similar to the TDP-43 mutant cell model, as previously reported (Fig. 4*A* and *SI Appendix*, Fig. S7 *D* and *E*) (34). Moreover, neurodegenerative features and disrupted nuclear membrane structures stained with Lamin-B1 were observed (*SI Appendix*, Fig. S8*A*).

To further identify Qki5 RNA targets that are linked to the molecular mechanisms underlying MN degeneration, we used SEEK analysis (Search-Based Exploration of Expression Compendium) (35) to identify genes that had strong coexpression correlations with *Qk* gene. Interestingly, we found the RNA targets of Qki5 in the top 20 genes (20%, five genes of the top 20 gene list, *SI Appendix*, Table S7). The number three gene was the *Map4k4* (HGK) gene, a JNK stress-related MAP kinase signaling gene. *Map4k4* encodes a serine/threonine kinase, which has been reported as a candidate therapeutic for ALS (36, 37). As we previously reported, *Map4k4* alternative splicing was dramatically changed in *Qk*-KD NSCs (38). We performed a loss-of-function study for *Map4k4* mRNA in mMNs and hiPSC-MNs. In *Qk*-KD cells, isoforms 1 and 3, which include exon 16, were up-regulated compared to the control. Therefore, Qki5 may promote exon 16 exclusion. We observed similar trends in mouse and human MNs (Fig. 4*B*).

In addition, the GO terms associated with the *Qk* gene in the SEEK top 200 gene list suggested the involvement of the “stress-activated MAPK pathway.” To examine the contribution of loss of Qki5 function to cell death-related JNK/SAPK signaling during neurodegeneration, we examined JNK pathway activation by immunostaining and Western blot analysis. We found enhancement of phosphorylated (P)-JNK and P-c-Jun staining in *siQk*-NSC-34 cells compared to *siNC* cells, even in no treatment with tumor necrosis factor-α (TNF-α). In addition, TNF-α-dependent activation of JNK and c-Jun was increased in *Qk*-KD cells (Fig. 4*C* and *SI Appendix*, Figs. S8 *B* and *C* and S9). Consistent with this, Western blot analysis showed upregulation of phosphorylated-TGF-β activated kinase (P-TAK), P-JNK, and P-c-Jun in *siQk*-NSC-34 cells at the basal level (0 min) and under TNF-α treatment, indicating that *Qk* KD caused overactivation of JNK/SAPK signaling. Interestingly, one of the downstream targets of JNK, γH2AX, was also significantly activated in *Qk*-KD cells (Fig. 4*D* and *SI Appendix*, Fig. S9, *P* = 0.013). Taken together, these results show that Qki5 contributes to the generation of the MN-specific transcriptome and safeguards MNs under cellular stress through pre-mRNA splicing.

### MN-Specific Deletion of *Qk* Causes MN Degeneration In Vivo.

**Fig. 5.**
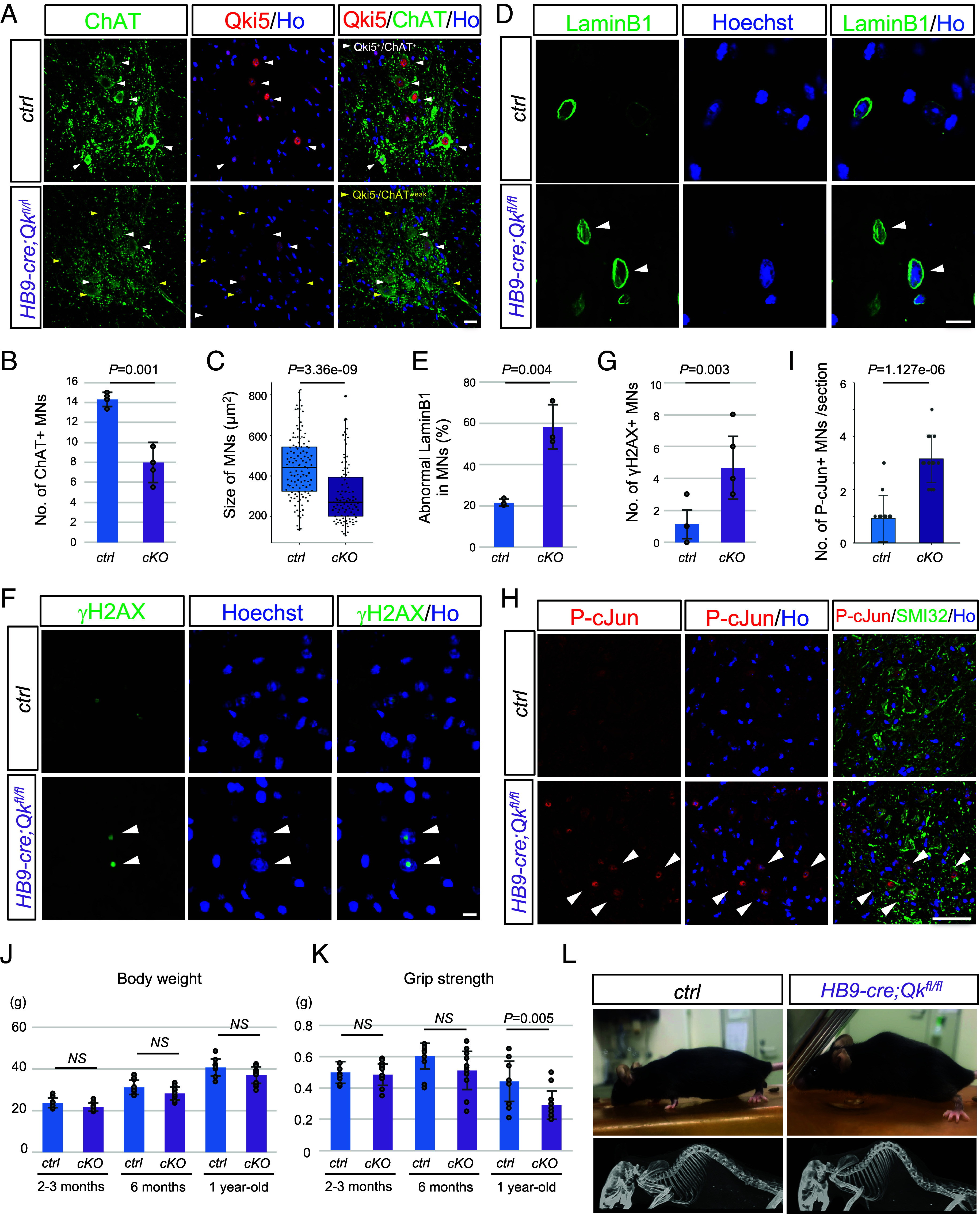
Qki5 deletion causes MN degeneration in the mouse spinal cord. (*A*) Double immunostaining analysis using antibodies against ChAT (green) and Qki5 (red) in control (*Qkfl/fl* or *Qkfl/+*) and *HB9-cre/+; Qkfl/fl* (cKO) spinal cords at 1 mo of age. Note that Qki5 expression was specifically down-regulated in the MN in cKO. White and yellow arrowheads indicate Qki5/ChAT double-positive and Qki5-negative and ChAT-weakly positive cells, respectively. (Scale bar, 20 μm.) (*B*) Bar graph showing the number of ChAT-positive MNs of the thoracic spinal cord was significantly decreased in 1-mo-old *HB9-cre/+; Qkfl/fl* mice compared to littermate controls (*Qkfl/fl* or *Qkfl/+*). Bars represent the mean ± SD for each group (mouse N = 4 each genotype). Dots represent for mean of MN number/section (N = 3 to 6 sections for each mouse), two-tailed Student’s *t* test. (*C*) Box blot showing the size of MNs in ctrl and cKO. The box represents the mean ± SD for each group (mouse N = 4 each genotype). Dots represent for the size for control; N = 160 MNs and cKO; N = 130 MNs- Welch two-sample *t* test. (*D*) The nuclear membrane was visualized by immunostaining with anti-LaminB1 antibodies (green). The nuclear membrane seemed to be disorganized within the nucleus. Arrowheads indicate abnormal nuclear membrane structures. (Scale bar, 10 μm.) (*E*) Bar graph showing the rate of aberrant structured laminB1 of MNs in ctrl and cKO. Bars represent the mean ± SD for each group (mouse N = 3 each genotype). Dots represent for mean of MNs with abnormal nuclear membrane structure of total MNs /section (N = 2 to 4 sections for each mouse, N = 88 MNs for ctrl and N = 78 for cKO). two-tailed Student’s *t* test. (*F*) Enhancement of the DNA damage response, as indicated by γH2AX immunostaining (green), was observed in Qki5-deleted MNs. (Scale bar, 10 μm.) (*G*) Bar graph showing the number of γH2AX-positive signals in MNs of the thoracic spinal cord in 1-mo-old *HB9-cre/+; Qkfl/fI* mice and littermate controls (*Qkfl/fl* or *Qkfl/+*). Data represent the mean ± SD, two-tailed Student’s *t* test. (*H*) Double immunostaining analysis using antibodies against SMI-32 (green) and Phospho-cJun (red) in control and cKO. (Scale bar, 50 μm.) (*I*) Bar graph showing the number of P-cJun-positive nuclei in SMI-32 positive MNs in 1-mo-old *HB9-cre/+; Qkfl/fI* mice and littermate controls (*Qkfl/fl* or *Qkfl/+*) section. Data represent the mean ± SD, two-tailed Student’s *t* test. (*J*) Bar graph indicates average body weight in male mice (*Left*, n = 13 for cKO mice and n = 9 for littermate controls) at 2 to 3 mo, 6 mo old, and 1 y old of age. Error bars show the SD, assessed with a two-tailed Student’s *t* test. (*K*) Bar graphs showing the grip strength in male mice shown in *F*. Error bars show the SD, assessed with a two-tailed Student’s *t* test. (*L*) Representative images (*Top*, 1 y old of mouse age) and CT scanning (*Bottom*, 1.5 y old of mouse age) for kyphosis assessment of cKO and littermate control mice.

Given that Qki5 have functions in MN cell lineage in vivo, we generated MN-specific *Qk* conditional KO (cKO) mice, which were obtained by crossing the *Qk*-flox mice with the *HB9*-cre driver line (15, 39). We first assessed the morphology and number of MNs in the thoracic spinal cord using IF analysis and observed that the Qki5 protein was still expressed in MNs in the E13.5 *HB9-cre; Qkflox/flox* cKO mice (*SI Appendix*, Fig. S10 *A* and *B*). After that, we confirmed that Qki5 protein was lost, which is specific to MNs in *HB9-cre;Qkflox/flox* spinal cord at 1 mo of age (Fig. 5*A*). In control mice, we observed healthy ChAT-positive MNs expressing Qki5 protein. On the other hand, the size of the cell body of ChAT-positive MNs seemed to be smaller, and the cytoplasm of MNs was found to be shrunken and degenerated at 1 mo of age (Fig. 5*A*). The number and size of MNs was significantly decreased in cKO mice compared to control mice at 1 mo of age (58.1% reduction and *P* = 0.001 for number of ChAT^+^ MNs; 31.0% reduction and *P* = 3.36e−09 for the size of MNs, n = 3 mice for each cKO and littermate control) (Fig. 5 *B* and *C*) (40–42). We assessed the lamin-B1-positive nuclear membrane structure was also disorganized in the remaining MNs, as observed in human TDP-43 proteinopathy (Fig. 5 *D* and *E* and *SI Appendix*, Fig. S11*A*) (43). Furthermore, γH2AX staining indicating the DNA damage response and cellular senescence, which is one of the common pathogenic mechanisms of neurodegeneration, such as FUS pathology, was significantly up-regulated in *Qk* cKO MNs (Fig. 5 *F* and *G*) (44). We next performed immunostaining with antibodies against P-c-Jun and P-TAK1, which is the downstream of Map4k4, and found that the number of MNs with P-c-Jun-positive nuclei was significantly increased in *Qk* cKO (Fig. 5 *H* and *I*), and that P-TAK1 intensity was up-regulated in *Qk* cKO compared to control (*SI Appendix*, Fig. S11 *B* and *C*). These results in *Qk* cKO are consistent with the in vitro culture model assay (Fig. 4). These data suggest that Qki5 deletion might cause cell biological abnormality, suggesting process of MN degeneration and cell vulnerability.

Next, we assessed body weight and motor function in *Qk* cKO mice. Body weight was shown to be a simple and reliable measure for disease onset and progression in the ALS model mouse (hSOD1 G93A) (45). The body weight of the *HB9-cre;Qkflox/flox* cKO mice tended to be reduced compared to that of the control. The cKO mice, however, did not show significant weight loss at any stage (Fig. 5*J* and *SI Appendix*, Fig. S11*D*). The average lifespan of *HB9-cre;Qkflox/flox* mice is likely to be normal. Next, we performed a bar-grip test to observe motor function. There was no difference in bar-grip scores between cKO and control mice at the age of 2 to 3 and 6 mo old, though histological changes were already observed in 1-mo-old cKO mice. In contrast, the bar-grip test showed that the grip strength of 1-y-old cKO mice was significantly lower than that of control mice (34.4% reduction, *P* = 0.005 for males, 41.4% reduction, *P* = 0.010 for females), suggesting muscle weakness (Fig. 5*K* and *SI Appendix*, Fig. S11*E*). Consistently with those, old cKO mice (8 of 9 cKO mice at the age of 1.5 y old) also exhibited thoracic kyphosis indicating muscle weakness, as confirmed by CT scanning (Fig. 5*L*). These symptoms related to motor function appeared at late adult stage symptom and it was similar to other MN disease models (46).

## Discussion

To our knowledge, the Qki5 protein is the only MN-specific RBP among neuronal population in the spinal cord. This Qki5 dominance among Qki isoforms in MNs is presumably controlled by molecular mechanisms, including autoregulation and nonautonomous regulation coupled with transcription and RNA cis-acting elements. For example, Qki5 is required for the efficient expression of all isoforms (Qki5/6/7) in C2C12 myotubes expressing predominantly Qki5 and regulates the expression of itself and Qki6 via the Qk-regulatory network (47). Alternatively, the RBP Rbfox2 reduces *QKI6* and *QKI7* expression by repressing alternative splicing of exons 7a and 7b, which encode QKI6 and QKI7 in human ESCs (48). Since Rbfox family proteins, including Rbfox2, are expressed in MNs and contribute to AS regulation (49), this might explain the undetectable expression of Qki6 and Qki7 in MNs. mRNA-seq analysis suggested that Qki5 is involved in the MN-specific transcriptome “MN-ness” through posttranscriptional regulation. Interestingly, the exon usage of the MN transcriptome was more similar to that of mOPCs than to that of CNs (Fig. 3*D*). This scenario could be due to Qki5 expression in MNs and glial cells, which importantly originate from common Olig2-positive stem/progenitor cells (50). Therefore, we believe this shared cell lineage origin could give rise to similar cell-type-specific transcriptomes in both cell lines through posttranscriptional regulation. We do not have a reasonable biological answer to why do MNs and glial cells share common alternative exon usages. A more detailed understanding of each alternative splicing event in MNs and OPCs vs. CNs might help elucidate the biological importance of this regulatory mechanism. Also, Feng et al. have shown the preferential expression of *Qk* in GABAergic interneurons (51). This might be the same analogy of cell fate determination between forebrain GABAergic interneurons and ventral spinal cord MNs. These two different types of neurons have the common feature of being produced from specific NSCs expressing the Olig2 transcription factor.

Transcriptome analysis using *Qk*-KD in mouse and human MNs indicated that there are a few genes with significant transcript level changes (Fig. 3*A*). Our study further showed that aberrant exon usage activates the NMD pathway and induces a subsequent decrease in the overall transcript level of genes, suggesting that the fundamental role of the Qki5 protein is alternative splicing regulation. In fact, we identified a large number of Qki5-dependent alternative splicing target genes in MNs and NSCs (15), in addition to another group’s reports in different cell types (14, 52, 53). Our previous study revealed that Qki5 plays a biological role in cell–cell interaction signaling in mouse embryonic NSCs (15). Kyoto Encyclopedia of Genes and Genomes (KEGG) pathway analysis using a list of Qki5 HITS-CLIP target genes indicated that Qki5 functions in the cell adhesion molecule pathway and tight junction pathway. A neuron is a specialized cell and has a very different function from a NSC, e.g., information transmission between neural circuits (54, 55). In this study, GO enrichment analysis using gene lists of Qki5-dependent AS changes in mouse MNs revealed synapse-related pathways, as well as cytoskeleton-related pathways (*SI Appendix*, Table S5). This phenomenon was more prominent for hiPSC-MNs (*SI Appendix*, Table S6). Interestingly, we found that the pathways associated with Qki5-dependent AS changes included synapse-related pathways such as splicing factor NOVA-regulated synaptic proteins in synapse formation in addition to cell junction organization (56). Therefore, Qki5 might play a fundamental role in a more specialized synaptic cell–cell interaction in the context of MNs, like cell–cell interactions in NSCs. Loss-of-function studies using MN-specific *Qk* ablation in mice showed a decrease in MN numbers and degeneration at a young adult age, suggesting that Qki5 is an essential factor for MN maintenance (Fig. 5). Indeed, genetic studies have strongly implicated alternative splicing in a growing list of human diseases (41, 57). Therefore, the accumulation of many aberrant alternative splicing events in loss-of-*Qk* function might cause the dysregulation of synaptic transmission, inducing MN vulnerability. Previous studies have reported that the MAP4K4 signaling cascade is involved in the activation of the downstream effectors JNK/SAPK and c-Jun and elevated phosphorylation of c-Jun-induced apoptosis in mouse MNs with trophic factor withdrawal (36). Small molecule screening identified an inhibitor of MAP4K4 activity that improves MN survival as a therapeutic target for ALS. We observed the constitutive activation of JNK/SAPK signaling and the phosphorylation of TAK1, JNK/SAPK, c-Jun, H2AX, and components of the TNF-α-induced JNK signaling cascade in *Qk*-KD mouse MNs (Fig. 4 *C* and *D*), suggesting MN vulnerability. Notably, phosphorylation of both H2AX and c-Jun was observed at the same time. Similarly, we further observed the increased number of MNs with P-cJun-positive nuclei and higher intensity of P-TAK1 in *Qk* cKO (Fig. 5 *H* and *I* and *SI Appendix*, Fig. S11 *B* and *C*). Thus, the failure of Qki-dependent posttranscriptional regulation might lead to JNK activation, phosphorylation of c-Jun and H2AX, and subsequent MN vulnerability. In humans, 6q terminal deletion syndrome is known to be a disease linked to the *QKI* gene. This syndrome is a terminal deletion of the long arm of chromosome 6, which contains a large group of genes. Defects of larger than 7.1 Mb will result in a defect containing a region of the *QKI* gene, named T-QKI, T-PRKN, T-MAP3K4, T-R showed a severe phenotype including infantile muscular hypotonia and hypoplasia of the corpus callosum, etc. These phenotypes, particularly hypoplasia of myelin, might involve haploinsufficiency of the *QKI* gene, but MN dysfunction on 6q syndrome remains unclear, even though it includes infantile muscular hypotonia (58, 59).

Our present study shows that the Qki5 protein is essential for defining a MN transcriptome to maintain MN-ness and safeguard MNs. Notably, Qki5 contributes to the maintenance of MNs through the posttranscriptional regulation and its dysfunction of this mechanism might cause MN vulnerability.

## Materials and Methods

### Mice.

All of the mouse procedures were performed in accordance with the guidelines of Niigata University. Briefly, homozygous *Qkflox/flox* animals (15) were intercrossed with *HB9(Mnx1)* cre knock-in mice (Strain #:006600, The Jackson Laboratory) (39, 60) to obtain motor-neuron-specific *Qk* conditional knockout mice. The day on which the vaginal plug was found was termed E0.5. Mouse tail DNA was genotyped by PCR with the specific primers *Qk*-loxF (5′-GAC ACC ATC TTT ACTT CCT G-3′) and *Qk*-loxR (5′-TGT CAA CCT ATT CGG GCA TT-3′), resulting in a band of 570 bp for the wild-type allele and 680 bp for the floxed allele. To detect the *HB9*-cre transgenes, the primers *HB9* wt_F (5′-TCT ACA GTT ATT CGG CAG CAG-3′), *HB9*mt_F (5′-TGA TTC CCA CTT TGT GGT TCT-3′), and *HB9* common_R (5′-CTG AGG GTA TGA GTA GGA AAG C-3′) were used, resulting in a band of 77 bp for the wild-type allele and 255 bp for the *HB9*-cre allele.

### Tissue Preparation and Immunohistochemistry.

One-month-old mice were deeply anesthetized with pentobarbital (40 to 50 mg/kg) intraperitoneally and transcardially perfused with ice-cold phosphate-buffered saline (PBS), followed by 4% paraformaldehyde (PFA) fixative in PBS. Mouse embryos were dissected from pregnant female mice, washed with ice-cold PBS and fixed with 4% PFA/PBS. Dissected spinal cords and mouse embryos were processed for immunostaining as described previously (61). The sections were boiled in 10 mM citric acid buffer (pH 6.0) for 5 min for antigen retrieval for all antibodies except for the anti-HB9 antibody. For double staining for HB9 and Qki5, sections were heated in 1 mM ethylenediamine tetraacetic acid (ETDA) for 20 min at 65 °C. Then, sections were blocked with 0.5% Blocking Reagent (B40932, Thermo Fisher) for 60 min at RT and incubated overnight at 4 °C with primary antibodies, followed by incubation with Alexa-dye conjugated secondary antibodies (Invitrogen 1:1,000). The primary antibodies used for immunohistochemistry are described in *SI Appendix*. For the detection of ChAT, primary antibodies were detected by using HRP-conjugated secondary antibodies (donkey anti-goat IgG, Jackson Laboratory, 1:500), and Alexa Fluor 488 TSA amplification (B40932, Thermo Fisher). The nuclei were stained with Hoechst 33258 (10 μg/mL, B2883, Sigma). Images of the immunostained specimens were collected using a confocal laser scanning microscope (FV1200: Olympus) and a BZ-X810 all-in-one microscope (KEYENCE).

### Micro–Computed Tomography (Micro-CT) Scanning.

Images were obtained using a micro-CT system (IVIS SpectrumCT In Vivo Imaging System (Caliper Life-Science, Hopkinton, MA, USA) with a high-resolution charge-coupled device/phosphor screen detector at Keio University School of Medicine.

### Human iPS Cell Culture and *QKI* Knockdown.

Human iPSCs (deidentified clone 201B7), obtained from iPS Portal, Inc. (Kyoto, Japan), were cultured and MNs were induced as described in Matsuo et al. (22). At 24 d in vitro (DIV) after induction of MNs, the cells were subjected to scRNA-seq analysis. For *QKI* knockdown experiments, hiPSC-MNs were formulated into lipid-based nanoparticles. Nanoparticulated siRNA was mixed with MNs at the final concentration of 12.5 nM. Seventy-two hours after transfection at 7 DIV induction of MNs from MN progenitors, cells were harvested and subjected to qRT-PCR and RNAseq analysis. siRNAs were purchased from Life Technologies. *siQKI*1-3 were previously used Assay ID s18083, 18084, and 18085.

### scRNA Sequence and Data Analysis.

Differentiated human MNs were dissociated into a single-cell suspension using Accutase (Innovative Cell Technologies). Dissociated cells were resuspended in Dulbecco's modified Eagle medium (DMEM)/F-12 medium (Thermo Fisher Scientific) containing 0.04% bovine serum albumin (BSA) at a concentration of 700 cells/μL (Target cell recovery: 3,000 cells per channel). Subsequently, the suspended cells were loaded onto a Chromium Single Cell 3′ Chip (10× Genomics) and processed through the Chromium Controller to generate single-cell gel beads in emulsion (GEMs). scRNA-seq libraries were prepared with the Chromium Single Cell 3′ Library & Gel Bead Kit v.2 (10× Genomics) according to the manufacturer’s protocol. Sequencing was performed by using the Illumina HiSeq System at Genewiz Inc. in read 1 (26 bp), i7 index (8 bp), read 2 (98 bp) configuration for three lanes (with 1% PhiX) to obtain actual output passing filter data as a final data. Details of data analysis are described in *SI Appendix*.

### RNA Sequence and Data Analysis.

Total RNA was obtained from three independent replicates of cultured cells. The RNA was extracted using QIAzol, followed by the generation of mRNA libraries using Illumina TruSeq protocols for poly-A selection, fragmentation, and adaptor ligation (Illumina, TruSeq RNA Sample Prep Kit v2). The multiplexed libraries were sequenced as 75-nt paired-end runs on an Illumina NextSeq 500 system. Sequence reads were mapped to the reference mouse and human genome (NCBI build 37.1/mm 10 and hg19) with OLego. Expression levels and alternative splicing events were quantified with the Quantas tool (23). Differential expression statistics of mRNA abundance were performed with EdgeR. Integrative Genomics Viewer was used to visualize alignments in mouse and human genomic regions.

### RT–PCR and Western Blots.

RT-PCR and quantitative RT–PCR analyses were performed as described elsewhere (3). At least three biological replicates were used for quantitative PCR with a StepOnePlus real-time PCR detection system (Applied Biosystems). The mouse *Gapdh* and human *GAPDH* genes were used as internal controls, and the results were calculated with the *ΔΔ*CT method. The PCR primers are described in *SI Appendix*. Sodium dodecyl sulfate-polyacrylamide gel electrophoresis (SDS–PAGE) and Western blots were performed using a standard procedure, as described in Hayakawa-Yano et al. (15). The membranes were blocked with 4% skim milk (Nacalai) in Tris Buffered Saline with Tween-20 (TBS-T) for 60 min at RT and incubated overnight at 4 °C with primary antibodies, followed by incubation with HRP-conjugated secondary antibodies (Southern Biotech, 1:10,000). To detect phosphorylated protein, membranes were blocked with 5% PhoshpoBLOCKER in TBS-T (AKR-103, Cell Biolabs). Signals were detected with Western Lightning Plus-ECL (Perkin Elmer) or SuperSignal West femto (Thermo Fisher) for signal enhancement.

## Supplementary Material

Appendix 01 (PDF)

## Data Availability

RNAseq data have been deposited in OPC/NSC34/hiPS-MN/scRNAseq (GSE218820-1, GSE218824, and GSE267791) (62–65). All other data are included in the article and/or *SI Appendix*.
