# Supplementary material for: Qki5 safeguards spinal motor neuron function by defining the motor neuron-specific transcriptome via pre-mRNA processing
Source: Proc Natl Acad Sci U S A. 2024 Sep 3;121(37):e2401531121. doi: 10.1073/pnas.2401531121 (PMC11406248; doi:10.1073/pnas.2401531121)
Supplement: Supplementary file 1 — Appendix 01 (PDF) [file pnas.2401531121.sapp.pdf]

Supplementary information

Qki5 safeguards spinal motor neuron function by defining the motor neuron-specific transcriptome via pre-mRNA processing

Yoshika Hayakawa-Yano, Takako Furukawa, Tsuyoshi Matsuo, Takahisa Ogasawara, Masahiro Nogami, Kazumasa Yokoyama, Masato Yugami, Munehisa Shinozaki, Chihiro Nakamoto, Kenji Sakimura, Akihide Koyama, Kazuhiro Ogi, Osamu Onodera, Hirohide Takebayashi, Hideyuki Okano, and Masato Yano*

*To whom correspondence may be addressed. Email: myano@med.niigata-u.ac.jp

Contents:

Supplementary Methods
Supplementary References
Supplementary Figures 1-11
Supplementary Table 1-7

Supplementary methods

Antibodies

The following primary antibodies were used for immunohistochemistry.

Rabbit anti-Qki5 (A300-183A, Bethyl, 1:1,000), mouse anti-Qki6 (N182/17, NeuroMab, 1:200), mouse anti-Qki7 (N182/15, NeuroMab, 1:200), mouse anti-Olig2 (MAB50, Millipore, 1:250), mouse anti-Islet1 (38.3F7, DSHB, 1:100), mouse anti-HB9 (81.5C10-c, DSHB, 1:200), human serum for Hu antigen (kindly provided from Dr. Darnell, 1:500), mouse anti-SMI-32 (non-phosphorylated NF-H, NE1023, Calbiochem, 1:500), goat anti-Choline acethyltransferase (ChAT, AB-144P, Millipore, 1:100-200), mouse anti-Lamin-B1 (66095-1-Ig, Protein tech, 1:1,000), mouse anti-γH2AX (Ab22551, Abcam, 1:500), mouse anti-RbFox3 (NeuN) (MAB377, Millipore, 1:500-1,000), mouse-anti-P-cJun(Ser73) (ab30620, 1:100), mouse-anti-GAD67(Chemicon, 1:200), anti-phosphorylated-TAK1 (S412, 9339, Cell Signaling Technology, 1:200).

The following primary antibodies were used for immunocytochemistry.
Rabbit anti-Qki5 (A300-183A, Bethyl, 1:500), mouse anti-SMI-32 (non-phosphorylated NF-H, NE1023, Calbiochem, 1:500), mouse anti-Lamin-B1 (66095-1-Ig, Protein tech, 1:1,000), rabbit anti-phosphorylated JNK (T183/Y185, 4668, Cell Signaling Technology, 1:400), rabbit anti-phosphorylated c-Jun (S63, 91952, Cell Signaling Technology, 1:400), anti-FUS/TLS (sc-47711, Santacruz, 1: 1,000), rabbit active-Capase-6 (3156-100, BioVision, 1:500).

The following primary antibodies were used for Wester blots: rabbit anti-Qki5 (A300-183A, Bethyl, 1:500), rabbit anti-phosphorylated-TAK1 (S412, 9339, Cell Signaling Technology, 1:1,000), rabbit anti-TAK1 (T5206 Cell Signaling Technology, 1:1,000), rabbit anti-phosphorylated-JNK (T183/Y185, 4668, Cell Signaling Technology, 1:1,000), rabbit anti-JNK (9252, Cell Signaling Technology, 1:1,000), rabbit anti-phosphorylated-c-Jun (S63, 91952T Cell Signaling Technology, 1:1,000), rabbit anti-c-Jun (9165, Cell Signaling Technology, 1:1,000), mouse anti-γH2AX (S139, Ab22551, Abcam, 1:500), and mouse anti-β-actin (A1978, Sigma, 1:4,000), α-tubulin (T9026, Sigma, 1:4,000).

Primers for RT-PCR and qRT-PCR

Primers used for PCR experiments were as follows:

For qRT-PCR;

mouse *L3hypdh* (142nt)

Forward: 5'-GGGCATTTTATGCGTTTGTTAGT-3'

Reverse: 5'-TCTTCACTCTCAGGATGGTTGA-3'

mouse *Daam2* (147nt)

Forward: 5'-ACCACCTCTCCCCTTTGATAG-3'

Reverse: 5'-AGTGGGGCTTCATTGCTGG-3'

mouse *Rab12* (156nt)

Forward: 5'-ACCTCAGCCTATTACAGAAGTGC-3'

Reverse: 5'-ACAGTCCAGCTTATTTCCAACC-3'

For RT-PCR;

mouse L3hypdh, 1st exon-extension (250/131nt)

Forward: 5'-GGTGACCGCCTTCGTAGAGT-3'

Reverse: 5'-TGTCCACCAGCACCTTTCC-3'

mouse *L3hypdh*, Intron-retain bet ex3 and ex4 (121/203nt)

Forward: 5'-TCTGCGTGTTTGCCGATG-3'

Reverse: 5'-CTTTTGAAGGCTCTGGTCTGGT-3'

mouse *Nrxn1* and human *NRXN1 (*141/117nt)

Forward: 5'-AGTGGAGCCTGTGAATGGAAA-3'

Reverse: 5'-GTGTAGCCCGTTGTGGTAAGAA-3'

mouse *Cask (*278/241/215/171nt)

Forward: 5'-CCGCACTCAGTCTTCGTCCTGTG-3'

Reverse: 5'-TTTTCCAGTTTACCCTGCCACCAG-3'

human *CASK (*278/241/215/171nt)

Forward: 5'-CCGCACTCAGTCTTCGTCCTGTG-3'

Reverse: 5'-TTTTCCAGTTTACCCTGCCACCAA-3'

human *NEK1* (213/129nt)

Forward: 5'-GCGAAAACGGGAAGCTATG-3'

Reverse: 5'-TCACCACGAAGTTTGGCTTT-3'

human *TAK1*(MAP3K7) (164/83nt)

Forward: 5'-TGAGGGCAAGAGGATGAGTG-3'

Reverse: 5'-TACGTCTTGGCTGTCCGTTG-3'

human *AGRN*(Y-exon) (135/123nt)

Forward: 5'-TGGACCAGGGTCTCACTGGA-3'

Reverse: 5’-GTAGAGCGGCTCCTTCAGGTT-3’

mouse *Map4k4 (*538/376/307/145nt)

Forward: 5’-ACCGAGCTTTCATGCTCCAGAG-3’

Reverse: 5’-TTGGCTATTCTGCTGTCCACCG-3’

human *MAP4K4 (*538/376/307/145nt)

Forward: 5’-GCCAAGCTTCCATGCTCCCGAG-3’

Reverse: 5’-CTGGCTATTCTGCTGCCCACTG-3’

mouse Daam2 Cyptic exon (116/145nt)

Forward: 5’-GCAGGTATTTCGGATTCTGGAC-3’

Reverse: 5’-CGTCTCTCACATTTCCTCTCTCTCT-3’

mouse *Rab1*2 Cyptic exon (318/128nt)

Forward: 5’-CCACCGTGGGTGTTGACTTT-3’

Reverse: 5’-GGCACTTCTGTAATAGGCTGAGGT-3’

Mouse OPC culture and *Qk* KD

OPCs were purified from mouse embryos (E14.5) using immunopanning, as described in Emery et al.,[1] and transfected with siRNA to inhibit *Qk* using days after siRNA transfection, the cells were collected and subjected to mRNA-seq.

HEK293-RFT cell culture and DOX-induced QKI expression

Full-length Qki5 coding regions were by subcloning into the pcDNA5/FRT/TO vector and co-transfection into Flp-In T-REx^{TM} HEK293 cells (Invitrogen) with Flp recombinase (encoding plasmid pOG-44). We cultured Flp-In T-REx^{TM} HEK293 cells in Dulbecco’s modified Eagle’s medium (DMEM, Gibco) supplemented with 10%

fetal bovine serum (FBS). For Flp-In T-REx^{TM} HEK293 selection, Hygromycin-resistant colonies were pooled and expanded. Transgene expression was induced with 1 μg/ml doxycycline (Sigma). X-tremeGene HP DNA transfection reagent (Roche) was used for transfections as previously described[2].

NSC-34 culture

NSC-34 cells were seeded onto collagen-coated (50 μg/mL, collagen type IV human placenta, 5533 MERCK) 24-well microplate or coverslips with 12mm of diameter in 24-well microplate. The normal medium was exchanged 24hrs after seeding to differentiation medium containing 1:1 D-MEM/Ham's F-12 (042-30555, Wako), 1% FBS (10270106, Thermo Fisher), 1% modified Eagle's medium nonessential amino acids (NEAA), 1% Penicillin-Streptomycin (168-23191, Wako), 5 μM retinoic acid (*all-trans* retinoic acid, R2625, MERCK) for 6 days. The differentiation medium was changed every two days. recombinant mouse TNF-α (PMC3014, Gibco or 201-13461, Fujifilm Wako) NSC-34 cells were maintained in general growth medium, and induction of differentiation was performed as described in Tebaldi et al.[3] with a slight modification. The cells were transfected with control siRNA or *Qk* siRNA at 4 days *in vitro* (DIV) and treated with or without 5 ng/ml TNF-α for the indicated periods on 6 DIV. Cells were treated with or without 100 μM Cycroheximide (C4859, Sigma) for 6 hrs and subjected for qRT-PCR.

Immunocytochemistry and SYTOX staining

NSC-34 cells were transfected with si-*Cont* or si-*Qk* at 4DIV. Fourty eight hours after transfection, cells were fixed with 4% PFA in PBS for 15 min for

immunocytochemistry and SYTOX green staining to detect the cell death (S34860, Thermo Fisher, 0.1mM). After washing with PBS, the cells were blocked with 0.5% Blocking Reagent for 60 min at RT and incubated overnight at 4 °C with primary antibodies, followed by incubation with Alexa dye-conjugated secondary antibodies (Invitrogen, 1:1000).

Cell lysis and Differential detergent fractionation

Cells were washed with ice-cold PBS and lysed with MAPK buffer (10 mM Tris-HCl pH 7.6, 150 mM NaCl, 30 mM sodium pyrophosphate, 50 mM sodium fluoride, 20 mM β-glycerophosphate, 1% Triton X-100 and a protease inhibitor mixture (Complete; 11697498001, Roche Applied Science). To analyze protein solubility, NSC-34 cells were lysed in MAPK buffer and sonicated with a Bioruptor II and centrifuged at 13,000 x g for 15 min at 4 °C. Then, the MAPK buffer-insoluble fraction was lysed in 1% Sarkosyl buffer (MAPK buffer containing 1% Sarkosyl instead of 1% Triton X-100), sonicated and centrifuged at 100,000 x g for 15 min at 4 °C. The Sarkosyl buffer-insoluble fraction was lysed with SDS sample buffer[4].

Behavioral tests

Twenty-two male mice (*n*=13 for cKO and *n*=9 for control) and female mice (*n*=7 for cKO and *n*=15 for control) were applied for time-course analysis of behavioral tests at 2-3 months, 6 months, and 1 year-old of age. Grip strength was measured by a grip strength meter (Ohara & Co.).

SEEK (Search-based Exploration of Expression Compendium) analysis

Gene co-expression analysis was performed using web-based co-expression search site (http://seek.princeton.edu/), Search-based Exploration of Expression Compendium[5]. For QKI gene query, we used multi-tissue profiling datasets for the default search result.

Counting of the number of motor neurons, quantification of signal intensity of Western blots and immunostaining, and statistics

To quantify the remaining motor neurons, we performed IF staining with anti-ChAT antibodies with Hoechst 33258 for 5 sections of the thoracic spinal cord for *Qk* cKO and control mice (n=3 for each group). The ChAT-positive neurons in the ventral horn were regarded as motor neurons. We examined the presence of ChAT immunoreactivity with a nucleus stained with Hoechst 33258 and calculated the number of remaining of motor neurons. The signal intensity of immunostaining on the indicated lines in images were measured by an RGB plot profile analysis using the ImageJ software program (NIH). Signal intensity quantification for Western blots was performed by using the ImageJ software program (NIH). All quantified data represent the average of the samples with the standard deviation (SD). Comparisons between the two groups were performed with unpaired two-tailed Student's t tests. A P value < 0.05 was considered statistically significant.

Gene ontology pathway enrichment analysis

Gene lists with alternative splicing changes between control and KD cells ($P<0.01$, $FDR<0.01$, $|DI|>0.05$ and $RPKM>1$) were used for pathway analysis. We conducted Gene Ontology (GO) and pathway analyses using the Metascape online platform

(http://metascape.org/).

Data analysis for scRNA sequence

Sequence reads were processed using Cell Ranger software v.7.0.1 (10x Genomics)[6], with reads mapping to human reference genome GRCh38/hg38. For quality control and most subsequent analyses, we used Asc-Seurat v.2.2.1 (Analytical single-cell Seurat-based web application), a comprehensive web application that encapsulates a series of tools for scRNA-seq data analysis[7]. Loupe Browser v6.3.0 (10x Genomics) was used to visualize the expression of genes at the cell level in the UMAP plots and the expression profile of genes in each cell cluster in the violin plots.

Data and materials availability

The datasets generated and analyzed in the current study are deposited in the Gene Expression Omnibus (GEO) repository under accession numbers GSE218820 (for OPCs), GSE218821 (for NSC-34 cells), GSE218824 (for hiPSC-MNs) and GSE267791 (for scRNAseq in hiPSC-MNs). Source data are provided with this paper. All data are available in the main text or the supplementary materials.

Supplementary References

1. Emery, B., and Dugas, J.C. (2013). Purification of oligodendrocyte lineage cells from mouse cortices by immunopanning. Cold Spring Harb Protoc. *2013*, 854-868. 10.1101/pdb.prot073973.

2. Koyama, A., Sugai, A., Kato, T., Ishihara, T., Shiga, A., Toyoshima, Y., Koyama, M.,

Konno, T., Hirokawa, S., Yokoseki, A. et al. (2016). Increased cytoplasmic TARDBP mRNA in affected spinal motor neurons in ALS caused by abnormal autoregulation of TDP-43. Nucleic Acids Res. *44,* 5820-5836. 10.1093/nar/gkw499.

3. Tebaldi, T., Zuccotti, P., Peroni, D., Köhn, M., Gasperini, L., Potrich, V., Bonazza, V., Dudnakova, T., Rossi, A., Sanguinetti, G. et al. (2018). HuD Is a Neural Translation Enhancer Acting on mTORC1-Responsive Genes and Counteracted by the Y3 Small Non-coding RNA. Mol Cell. *71,* 256-270.e10. 10.1016/j.molcel.2018.06.032.
4. Iguchi, Y., Katsuno, M., Takagi, S., Ishigaki, S., Niwa, J., Hasegawa, M., Tanaka, F., and Sobue, G. (2012). Oxidative stress induced by glutathione depletion reproduces pathological modifications of TDP-43 linked to TDP-43 proteinopathies. Neurobiol Dis. *45,* 862-870. 10.1016/j.nbd.2011.12.002.
5. Zhu, Q., Wong, A.K., Krishnan, A., Aure, M.R., Tadych, A., Zhang, R., Corney, D.C., Greene, C.S., Bongo, L.A., Kristensen, V.N. et al. (2015). Targeted exploration and analysis of large cross-platform human transcriptomic compendia. Nat Methods. *12,* 211-4, 3 p following 214. 10.1038/nmeth.3249.
6. Zheng, G.X., Terry, J.M., Belgrader, P., Ryvkin, P., Bent, Z.W., Wilson, R., Ziraldo, S.B., Wheeler, T.D., McDermott, G.P., Zhu, J. et al. (2017). Massively parallel digital transcriptional profiling of single cells. Nat Commun. *8,* 14049. 10.1038/ncomms14049.
7. Pereira, W.J., Almeida, F.M., Conde, D., Balmant, K.M., Triozzi, P.M., Schmidt, H.W., Dervinis, C., Pappas, G.J., and Kirst, M. (2021). Asc-Seurat: analytical single-cell Seurat-based web application. BMC Bioinformatics. *22,* 556. 10.1186/s12859-021-04472-2.

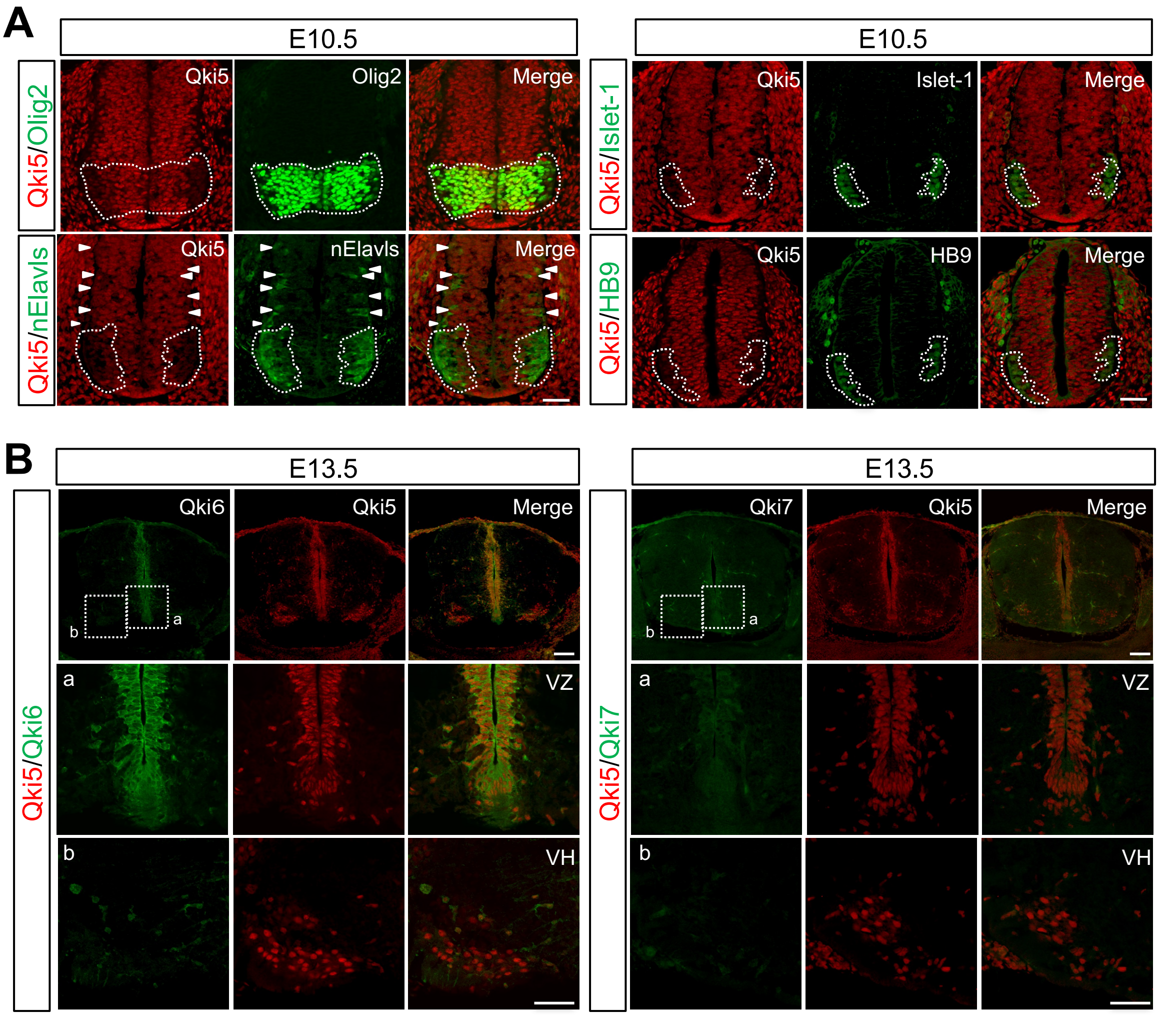

Fig. S1. Qki protein expression in the motor neuron lineage.
A, Detailed expression of Qki5 and motor neuron (MN)-lineage marker proteins in the E10.5 mouse spinal cord. Qki5 (red) colocalizes with the progenitor marker Olig2 (green, surrounded by dashed lines) in the pMN domain of the ventricular zone (top left panel). In contrast, Qki5 expression is not observed in nElavls-positive neuronal population (green, surrounded by dashed lines and arrowheads) and MNs labeled with Islet-1 (green, middle) or HB9 (green, bottom) at this timepoint. Scale bar, 50 μm.
B, Double immunostaining for Qki5 (red) and Qki6 or Qki7 (green). Qki5 is expressed in somatic and visceral motor neurons and in neural progenitors at E13.5. Neither Qki6 nor Qki7 was detectable in the motor neuron population, while Qki6 was expressed only in neural progenitors in the ventricular zone (VZ) and oligodendrocyte progenitors (OPC). Panels a and b represent the enlarged views of VZ and VH (ventral horn) indicated by the insets in top-left panels, respectively. Scale bars: 100 μm for low-magnification images and 50 μm for insets.

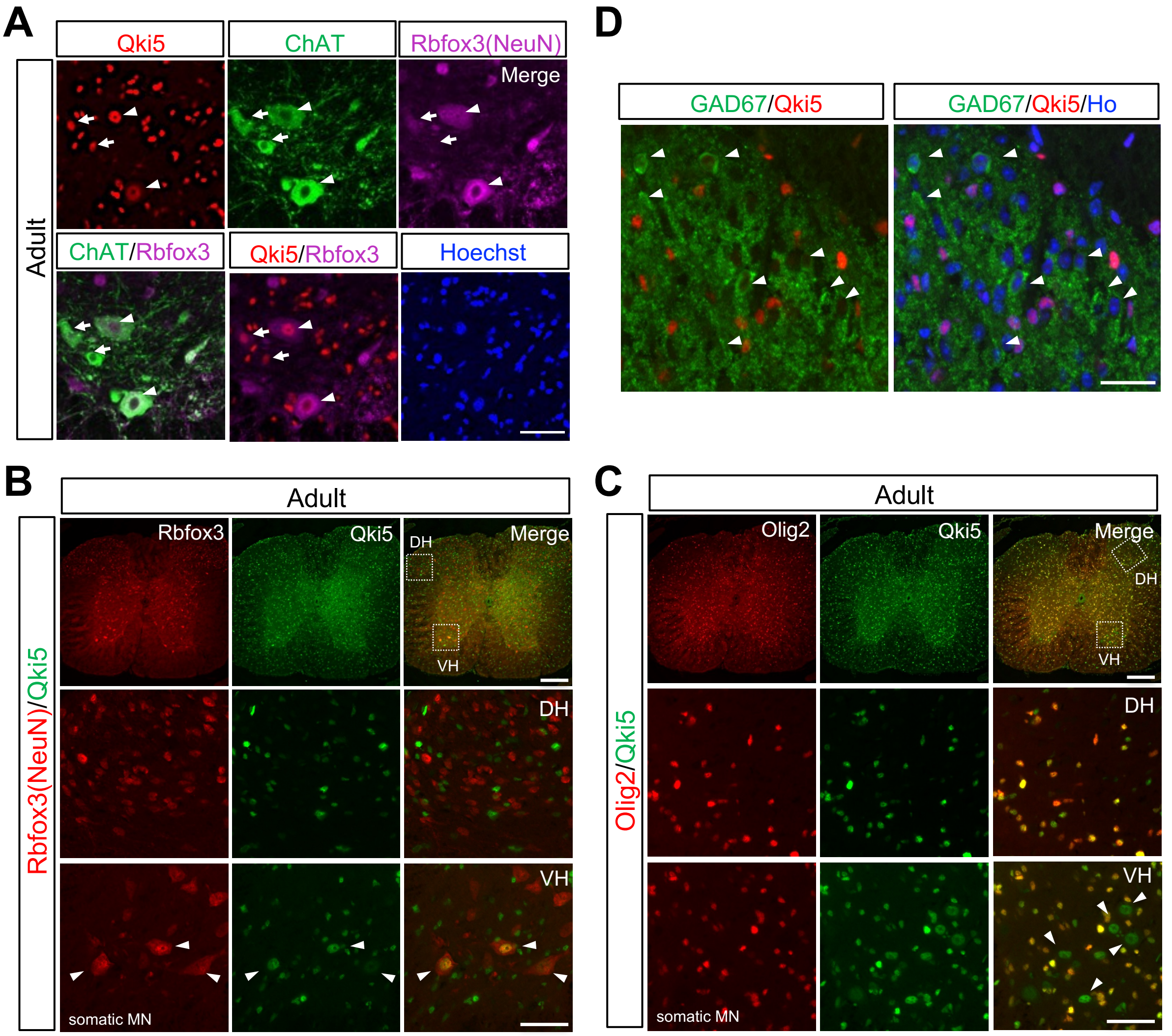

Fig. S2. Qki5 expression in motor neuron subtypes.

A, Triple immunostaining for Qki5 (red), ChAT (green) and RbFox3 (NeuN, purple) in the mouse spinal cord at 1 month of age. Qki5 expression was detectable in both ChAT$^+$/Rbfox3+ α-motor neurons (indicated by arrowheads) and ChAT$^+$/Rbfox3$^{-/weak}$ γ-motor neurons (indicated by arrows). Scale bar: 20 μm.

B, Immunostaining for Rbfox3 (NeuN, red) and Qki5 (green) of transverse sections in mouse spinal cord at 1 month of age. Lower panels represent the enlarged views of the areas indicated by the insets in top panel, respectively. Arrowheads represent the Qki5 expression in NeuN–positive neurons in ventral horn (VH), but not in dorsal horn (DH). Scale bars: 200 μm for low-magnification images and 50 μm for insets.

C, Immunostaining for Olig2 (red) and Qki5 (green) of transverse sections in mouse spinal cord at 1 month of age. Lower panels represent the enlarged views of the areas indicated by the insets in top panel, respectively. Qki5 is expressed in both Olig2-negative MNs (indicated by arrowheads) and Olig2-negative OLs). Scale bars: 200 μm for low-magnification images and 50 μm for insets.

D, Immunostaining for Qki5 (red) and GAD67 (green) of transverse sections in mouse spinal cord (Dorsal Horn) at 1 month of age. Arrowheads represent Qki5 is not expressed in GAD67-positive GABAergic neurons. Scale bar: 50 μm

E

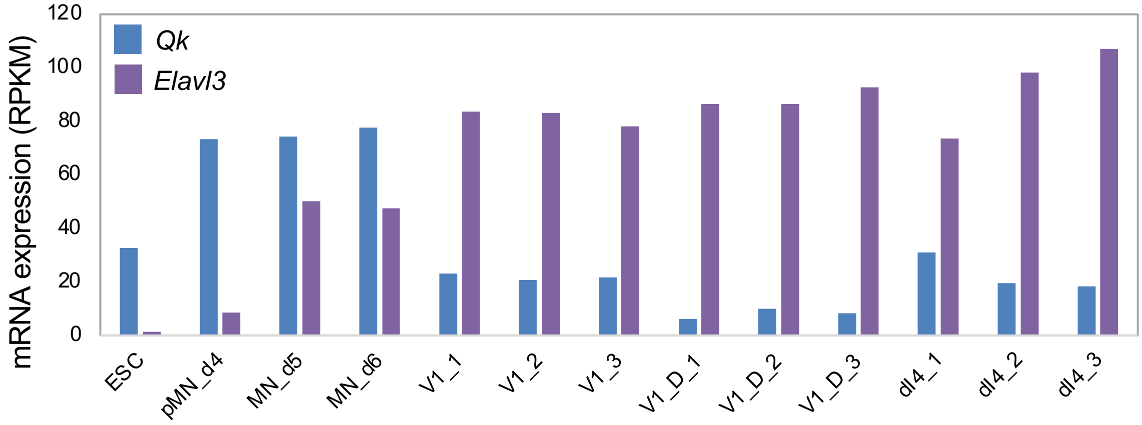

F

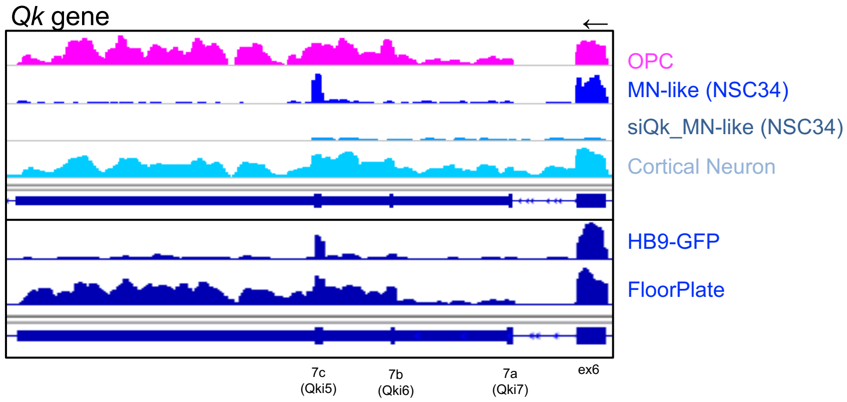

Fig. S2. Qki5 expression in motor neuron subtypes.

E, Reanalysis from public RNA-seq datasets from GSE112377 and GSE79561 confirmed that *Qk* transcript is enriched in the ESC-derived MNs, relative to other types of neurons, ESC-derived V1 and V1D and dI4 neurons. On the other hands, *Elavl3* transcript, encoding an another neuronal RBP is widely expressed in multiple types of neurons.

F, IGV image of alternative splicing changes in the *Qk* gene from RNA-seq data including this paper, GSE52564 and GSE75601. 7c exon (Qki5) is predominantly expressed in HB9-GFP motor neurons and NSC-34 cells.

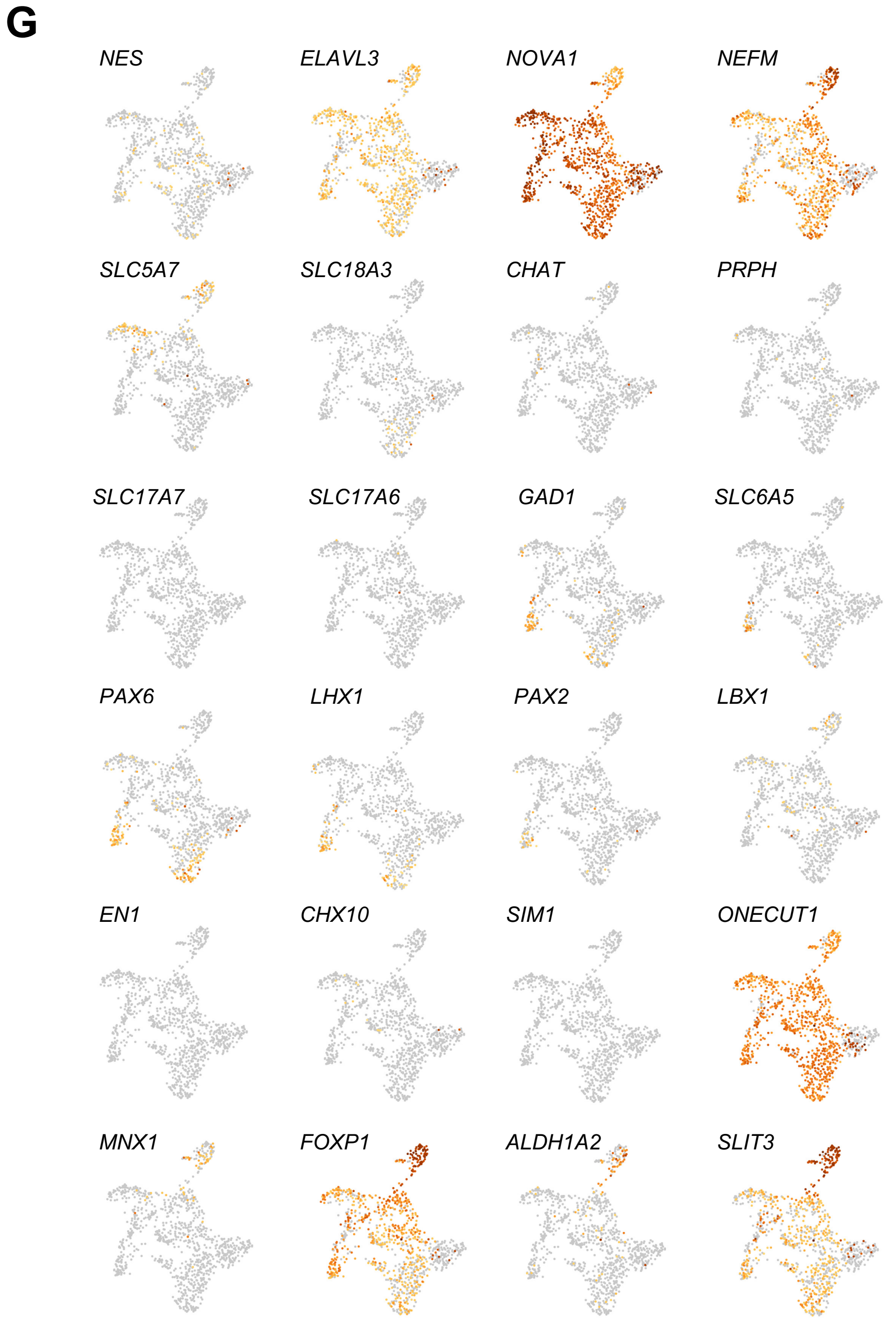

Fig. S2. Qki5 expression in motor neuron subtypes.
G, Representative UMAP plots of single-cell RNA-seq using hiPSC-MNs showing additional 24 gene expression in 6 clusters related to Fig. 2B.

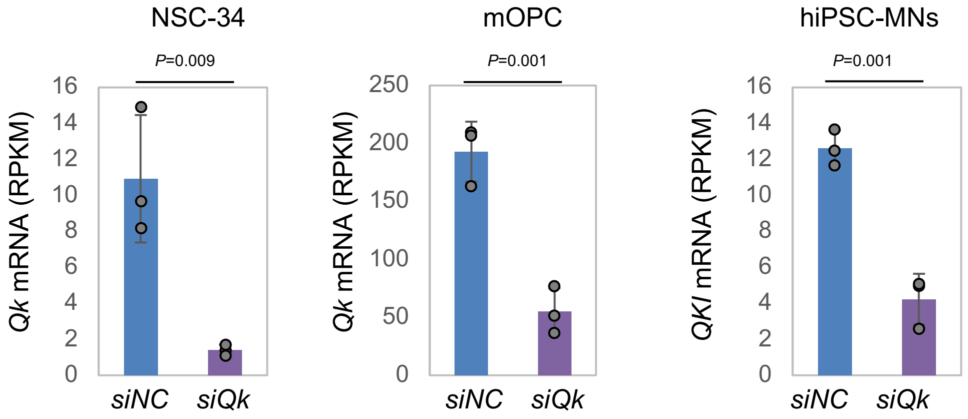

Fig. S3. Knock-down efficiency in three different cultured cell model.
mRNA sequence confirmed that downregulation of *Qk* or *QKI* transcript in the knock-down cells. Bar graph for the level of *Qk* transcript in NSC-34 cells and mOPC and *QKI* transcript in hiPSC-MNs, respectively. Data represents the mean from in three-independent biological replicates transfected with three different siRNAs. Data represent the mean ± SD of three independent experiments. Two-tailed Student's *t*-test.

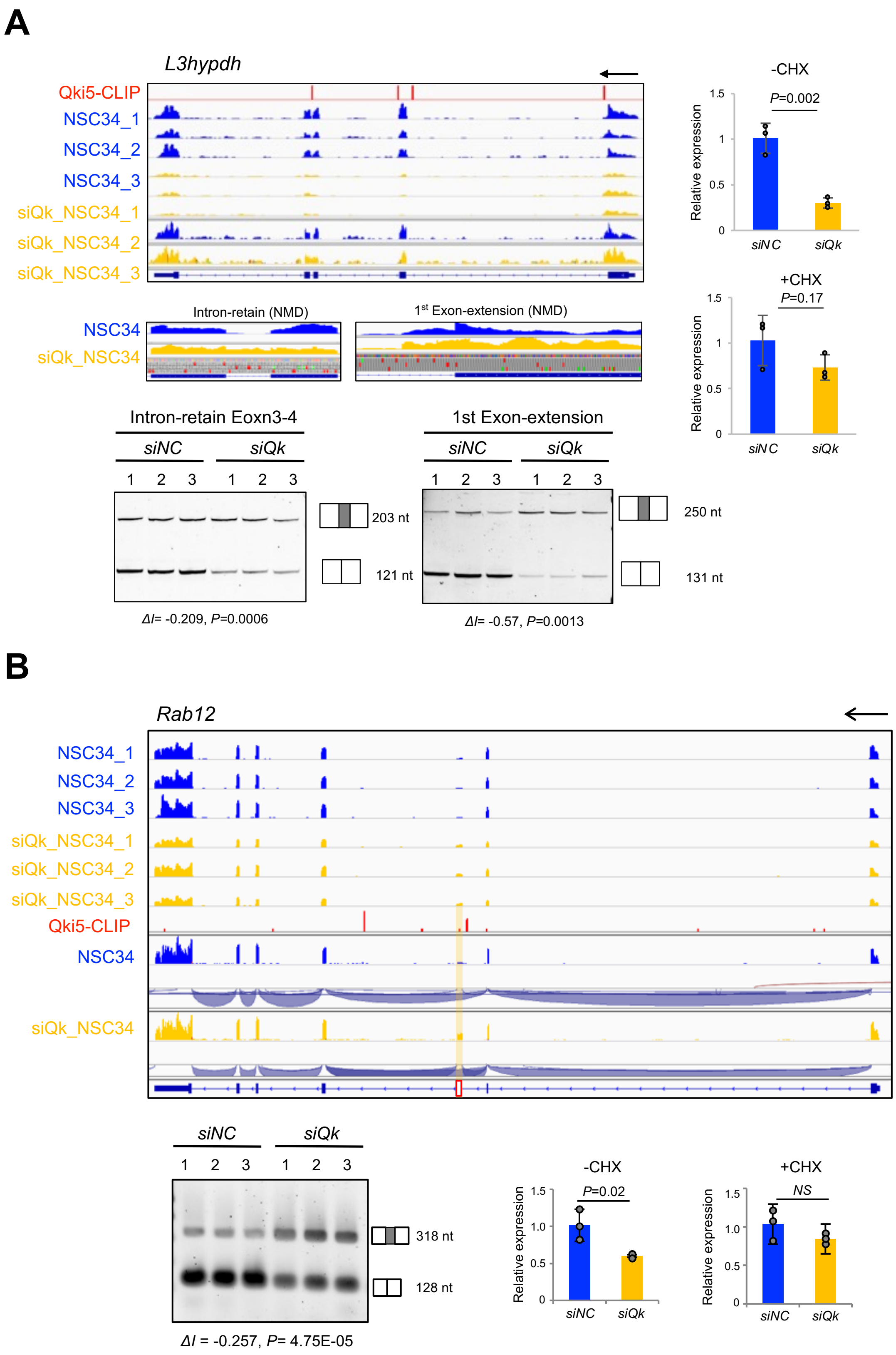
A
L3hypdh
Qki5-CLIP
NSC34_1
NSC34_2
NSC34_3
siQk_NSC34_1
siQk_NSC34_2
siQk_NSC34_3
Intron-retain (NMD)
1st Exon-extension (NMD)
NSC34
siQk_NSC34
Intron-retain Eoxn3-4
siNC
siQk
1 2 3 1 2 3
203 nt
121 nt
ΔI= -0.209, P=0.0006
1st Exon-extension
siNC
siQk
1 2 3 1 2 3
250 nt
131 nt
ΔI= -0.57, P=0.0013
-CHX
P=0.002
Relative expression
1.5
1
0.5
0
siNC
siQk
+CHX
P=0.17
Relative expression
1.5
1
0.5
0
siNC
siQk
B
Rab12
NSC34_1
NSC34_2
NSC34_3
siQk_NSC34_1
siQk_NSC34_2
siQk_NSC34_3
Qki5-CLIP
NSC34
siQk_NSC34
siNC
siQk
1 2 3 1 2 3
318 nt
128 nt
ΔI = -0.257, P= 4.75E-05
-CHX
P=0.02
Relative expression
1.5
1.0
0.5
0
siNC
siQk
+CHX
NS
Relative expression
1.5
1.0
0.5
0
siNC
siQk

C

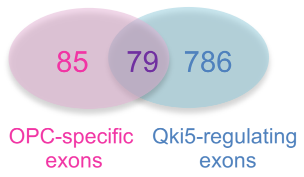

D

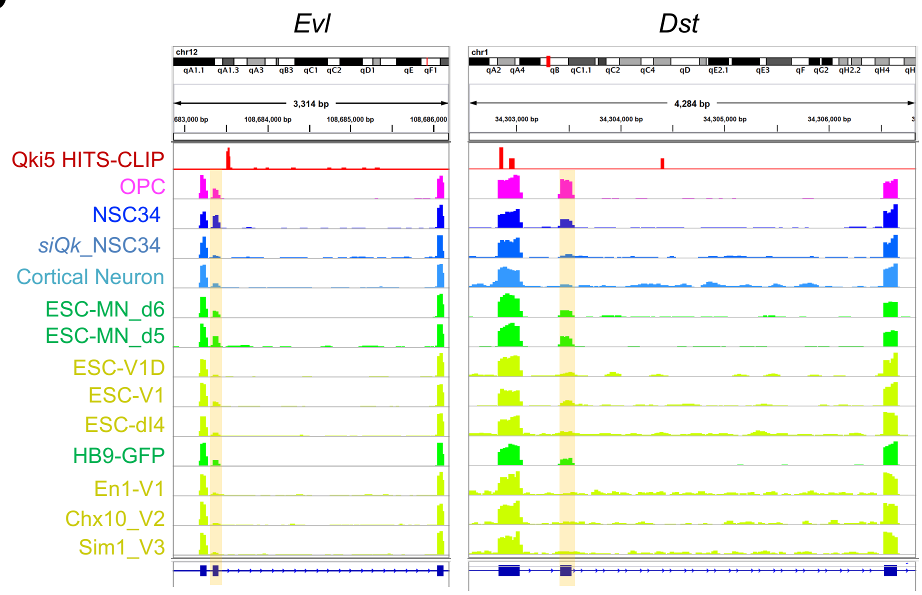

Fig. S4. Qki5 loss causes a decrease in transcript levels through alternative splicing-dependent nonsense-mediated decay.

A, Representative IGV view of alternative splicing changes in the *L3hypdh* gene in *siNC* and *siQk* NSC-34 cells with Qki5 HITS-CLIP clusters. *siQk* cells show that 1st exon extension and intron retention induce nonsense-mediated decay (NMD). Gel images for RT–PCR validation of intron retention between exon 3 and exon 4 (bottom-left) and 1st exon extension (bottom-right). Bar graphs indicate the qRT-PCR assay of the relative expression of *L3hypdh* transcript adjusted to the internal control *Gapdh* using *siNC* and *siQk* NSC-34 cells with or without cycloheximide (CHX) treatment. Data represent the mean ± SD. from three independent biological replicates. Two-tailed Student's *t*-test.

B, IGV image of alternative splicing changes in the *Rab12* gene. Gel images of the RT–PCR validation assay show the inclusion of cryptic exons between exons 2 and 3, inducing NMD. qRT–PCR assays indicated that CHX treatment restored transcript levels (right). Bar graphs indicate the relative expression levels of *Rab12* adjusted according to the internal control *Gapdh* gene. Data represent the mean ± SD. from three independent biological replicates, assessed with a two-tailed Student's *t*-test.

C, Venn diagram showing the overlap of the two datasets and the numbers of OPC-specific exons and Qki5-regulated exons.

D, Qki5 regulates alternative splicing of *Evl and Dst* gene in Qki5 expressing cells. IGV showing the mRNA expression level along the two genes from RNA-seq data including this paper, GSE112377, GSE79561 and GSE112377. The expression level of alternative exons (highlighted in yellow) might correlate with Qki5 protein expression in those cells.

A

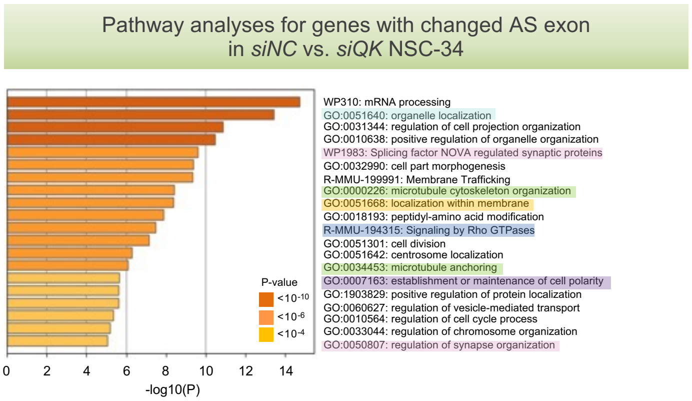

B

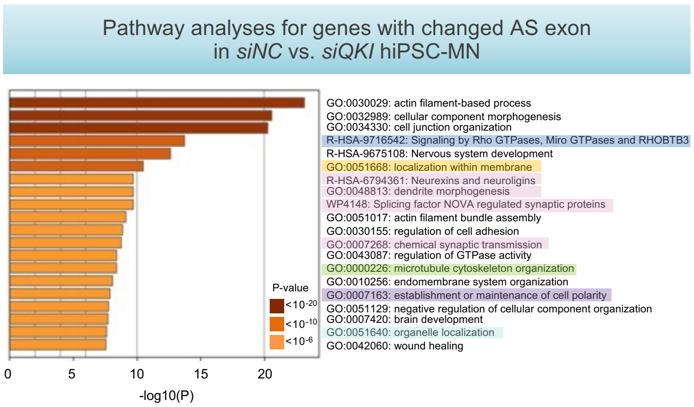

Fig. S5. Enrichment pathway analyses reveal that Qki5-dependent posttranscriptional regulation contributes to cytoskeleton organization and synapse formation

A-B, Enrichment pathway analyses using a gene list with 660 changed alternative splicing (AS) exons in *siNC* vs. *siQk* NSC-34 cells (cutoff: P<0.01, FDR<0.1 and |DI|>0.05) in **A** and 388 changed AS exons in *siNC* vs. *siQKI* hiPSC-MNs (cutoff: P<0.01, FDR<0.01 and |DI|>0.05) in **B**. The figures were obtained from gene ontology analysis using the Metascape database (https://metascape.org/gp/index.html#/main/step1). The enrichment bar graph shows that the top 20 enriched terms (including GO terms, Reactome pathways, and Wiki pathways) with log10(p-value) are indicated on a heatmap. The same color highlights the common enriched pathways between NSC-34 cells and hiPSC-MNs.

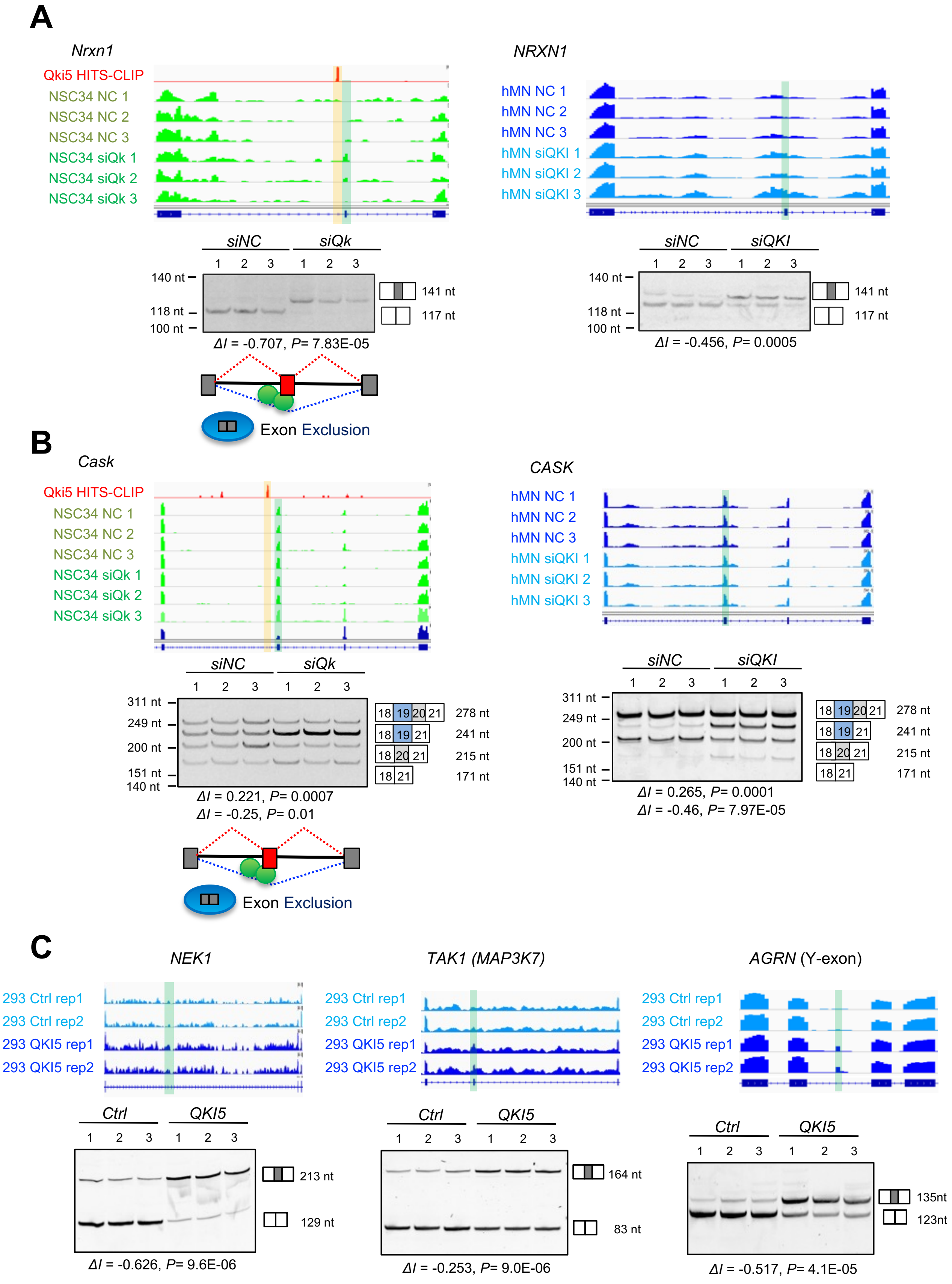
A
Nrxn1
Qki5 HITS-CLIP
NSC34 NC 1
NSC34 NC 2
NSC34 NC 3
NSC34 siQk 1
NSC34 siQk 2
NSC34 siQk 3
siNC
siQk
1 2 3 1 2 3
140 nt
118 nt
100 nt
141 nt
117 nt
ΔI = -0.707, P= 7.83E-05
Exon Exclusion
NRXN1
hMN NC 1
hMN NC 2
hMN NC 3
hMN siQKI 1
hMN siQKI 2
hMN siQKI 3
siNC
siQKI
1 2 3 1 2 3
140 nt
118 nt
100 nt
141 nt
117 nt
ΔI = -0.456, P= 0.0005
B
Cask
Qki5 HITS-CLIP
NSC34 NC 1
NSC34 NC 2
NSC34 NC 3
NSC34 siQk 1
NSC34 siQk 2
NSC34 siQk 3
siNC
siQk
1 2 3 1 2 3
311 nt
249 nt
200 nt
151 nt
140 nt
18 19 20 21 278 nt
18 19 21 241 nt
18 20 21 215 nt
18 21 171 nt
ΔI = 0.221, P= 0.0007
ΔI = -0.25, P= 0.01
Exon Exclusion
CASK
hMN NC 1
hMN NC 2
hMN NC 3
hMN siQKI 1
hMN siQKI 2
hMN siQKI 3
siNC
siQKI
1 2 3 1 2 3
311 nt
249 nt
200 nt
151 nt
140 nt
18 19 20 21 278 nt
18 19 21 241 nt
18 20 21 215 nt
18 21 171 nt
ΔI = 0.265, P= 0.0001
ΔI = -0.46, P= 7.97E-05
C
NEK1
293 Ctrl rep1
293 Ctrl rep2
293 QKI5 rep1
293 QKI5 rep2
Ctrl
QKI5
1 2 3 1 2 3
213 nt
129 nt
ΔI = -0.626, P= 9.6E-06
TAK1 (MAP3K7)
293 Ctrl rep1
293 Ctrl rep2
293 QKI5 rep1
293 QKI5 rep2
Ctrl
QKI5
1 2 3 1 2 3
164 nt
83 nt
ΔI = -0.253, P= 9.0E-06
AGRN (Y-exon)
293 Ctrl rep1
293 Ctrl rep2
293 QKI5 rep1
293 QKI5 rep2
Ctrl
QKI5
1 2 3 1 2 3
135nt
123nt
ΔI = -0.517, P= 4.1E-05

Fig. S6. Qki5 regulates synapse-related molecules via pre-mRNA splicing in mouse and human motor neurons

A, Qki5-dependent AS regulation of mouse *Nrxn1* and human *NRXN1*. Schematic representation of Qki5 binding position-dependent alternative exon usage (cassette exon) in the top panel. IGV image of Qki5 HITS-CLIP clusters and expression levels of the transcript in *siNC* and *siQk* NSC-34 cells with three independent biological replicates in the left top panel and expression levels of the transcript in *siNC* and *siQk* hiPSC-MNs in the right top. In the case of Nrxn1, Qki5 HITS-CLIP clusters were found upstream and on alternative exon 7 of the transcripts and inhibited exon usage (indicated by alternative splicing of SS2A). RT–PCR validation assays were performed to monitor the effect of Qki5 on the *Nrxn1* transcript, indicating Qki5-dependent exclusion in both mouse *Nrxn1* and human *NRXN1* (bottom).

B, Qki5-dependent AS regulation for *CASK*, as shown in (A). IGV image showing that the mouse *Cask* gene harbors two tandem alternative exons (19 and 20), and Qki5 HITS-CLIP clusters were found upstream of alternative exon 19 in the left top panel. Human *CASK* also has a similar exon–intron structure to the mouse gene (right top). Gel images of the RT–PCR validation assay for mouse *Cask* and human *CASK* (bottom). The exon 19-including form (exons 18-19-21) and exon 19 and 20 double-negative form (exons 18-21) were upregulated in both mouse and human *Qk-KD* cells.

C, AS changes in motor neurons or MND-related molecules in inducible-QKI5-expressing HEK293 cells. IGV images showing *NEK1*, *TAK1* (*MAP3K7*), and *AGRN* transcripts in control and QKI5-overexpressing cells with two biological replicates (top). Gel images of the RT–PCR validation assay confirmed that QKI5 inhibits exon inclusion of these alternative exons (bottom).

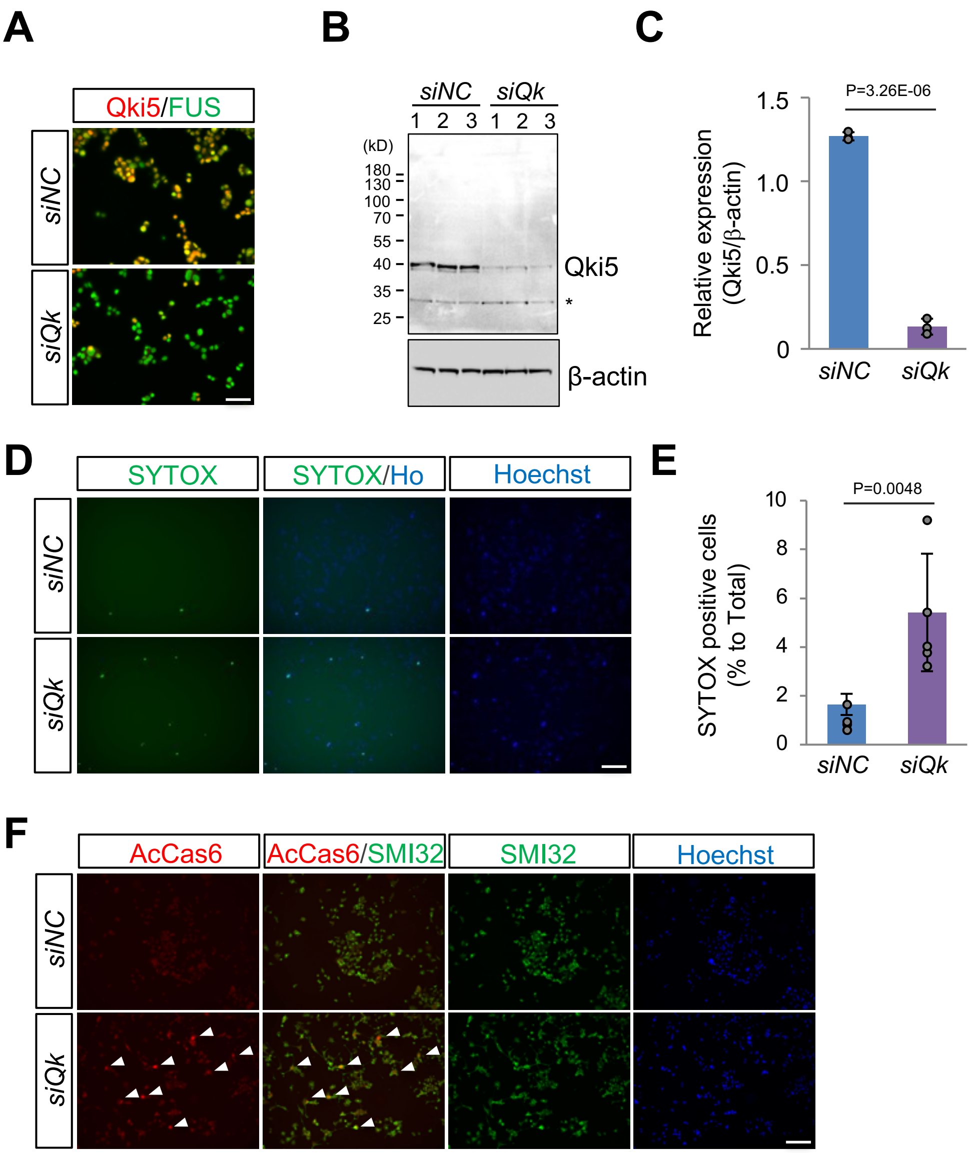

Fig. S7. Verification of reduced Qki5 protein levels and enhanced cell death marker levels in *Qk-KD* cells.

A, Immunocytochemistry using antibodies against Qki5 (red) and FUS (green) in *siNC*- or *siQk*-transfected NSC-34 cells. Scale bar, 50 μm.

B, Western blots using antibodies against Qki5 and β-actin in *siNC*- or *siQk*- transfected NSC-34 cells. Asterisk represents non-specific band that can also appear in *Qk* KD cells

C, Values were normalized to β-actin to obtain relative densitometric intensity of Western blot shown in **B**. Data represent the mean ± SD of three independent experiments. Two-tailed Student's *t*-test.

D, NSC-34 cells were transfected with *siNC* or *siQk* and then stained for SYTOX-green to detect dead cells 48 hours after transfection. Scale bar, 200 μm.

E, Bar graphs indicate the percentage of SYTOX-positive cell number in Hoechst-positive cell. Data represent the mean ± SD of three independent experiments. Two-tailed Student's *t*-test.

F, Immunocytochemistry using antibodies against active Caspase-6 (red) and SMI-32 (green). Arrowheads represent active Caspase-6-positive cells in *Qk-KD* cells. Scale bar, 200 μm.

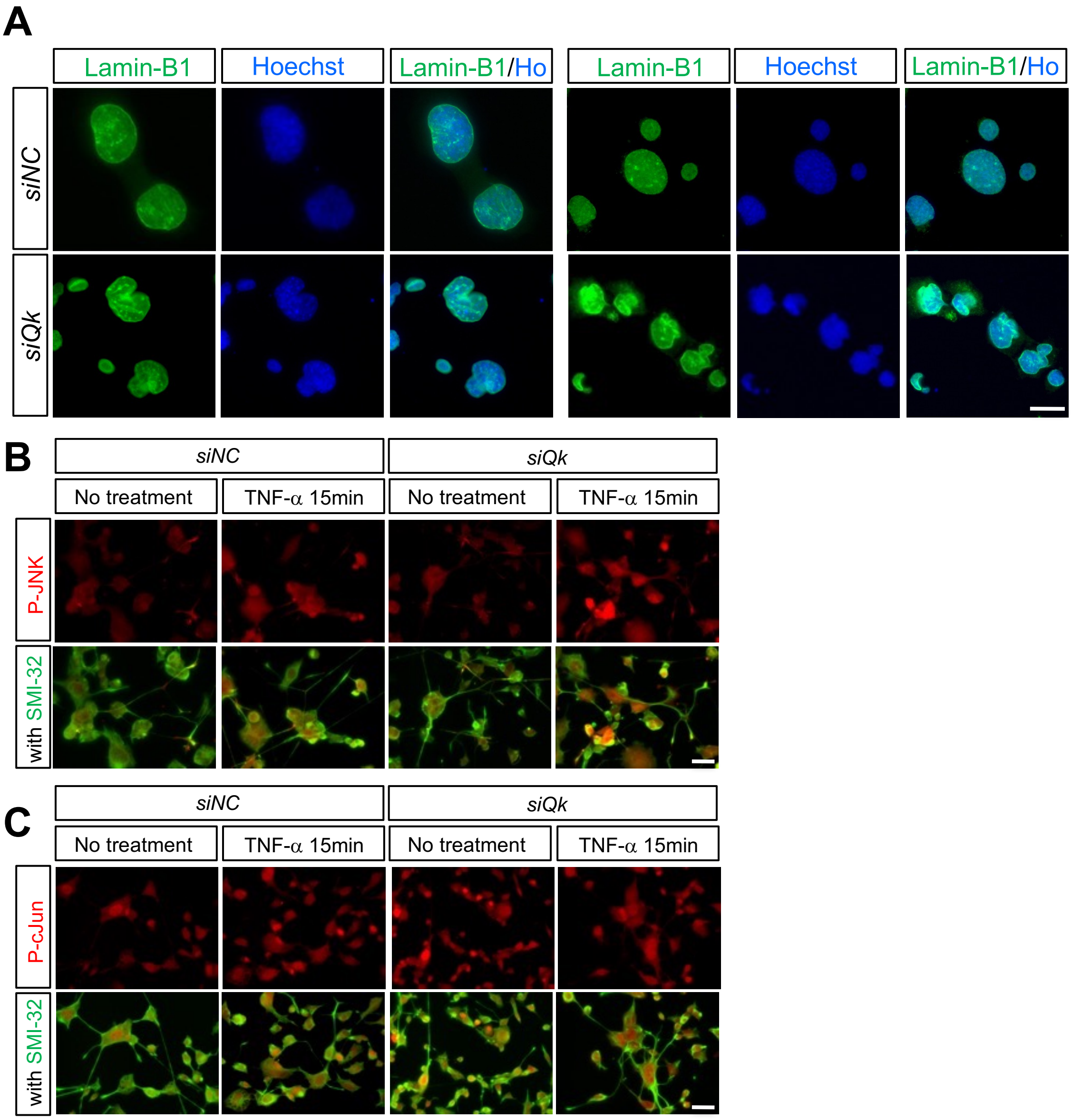

Fig. S8 *Qk-KD* resulted in the disruption of nuclear membrane structure and activation of JNK signaling pathway.

A, *Qk-KD* resulted in shrinkage of the nucleus, with a disorganized nuclear membrane structure labeled by Lamin-B1 staining (green). Scale bar, 25 μm.

B, Differentiated NSC-34 cells were transfected with *siNC* or *siQk*. Two days after transfection, the cells were treated with TNF-α or DMSO for 15 min and fixed for immunostaining. Double immunostaining using a phospho-specific JNK antibody (Thr183/Tyr185) (red) and SMI-32 (green) showed that TNF-α-induced JNK activation was upregulated in *Qk-KD* cells. Scale bar, 50 μm

C, Constitutive (no treatment) and TNF-α stimulation-dependent phosphorylation of c-Jun (Ser63) (red) in *Qk-KD* cells was increased compared to that in control cells. Additionally, SMI-32-positive inclusions in neurites were upregulated in *Qk-KD* cells compared to *siNC* cells under both untreated and TNF-α-treated conditions. Scale bar, 50 μm.

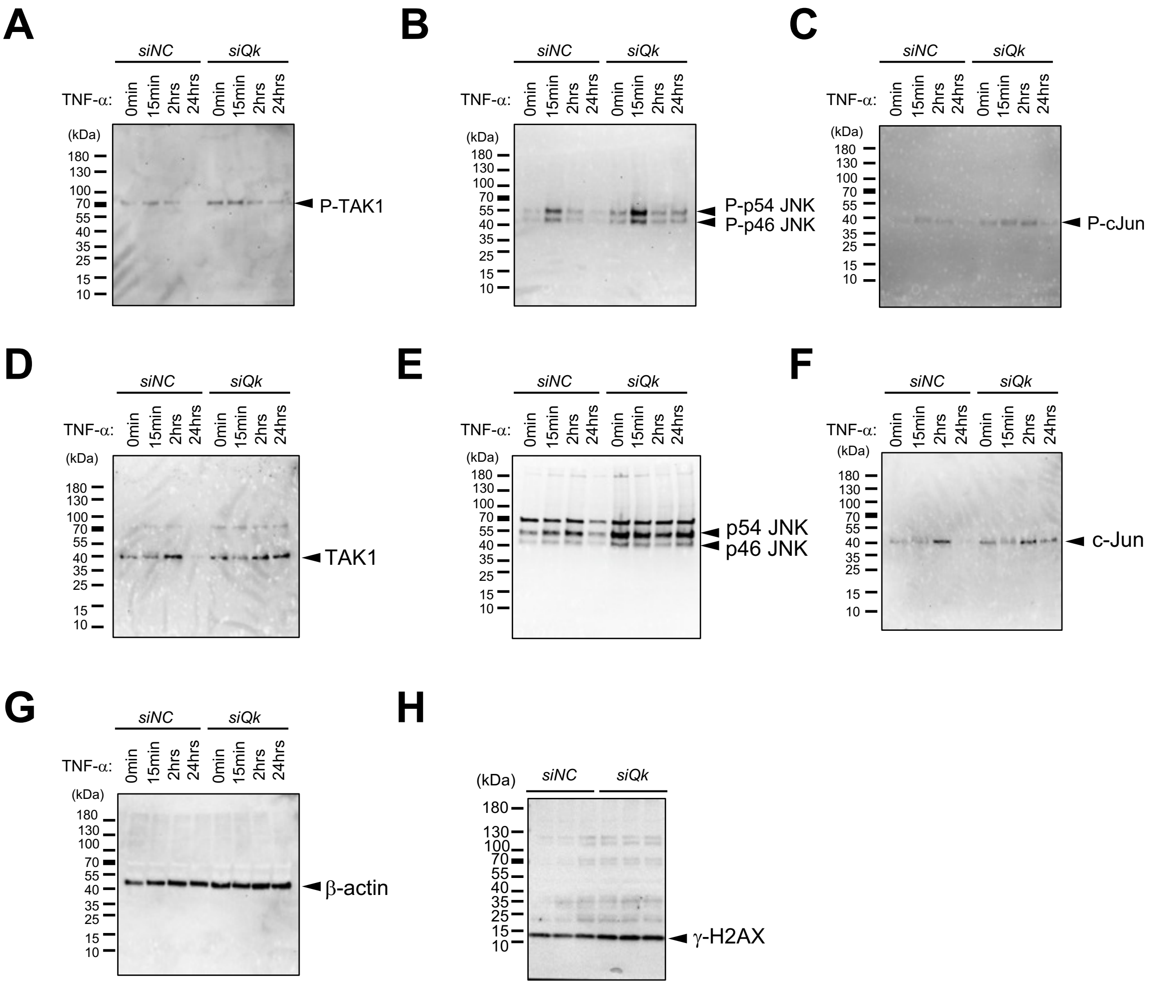

Fig. S9. Western blot analyses for the activation of JNK/SAPK and DNA-stress response pathway in *siNC* and *siQk* cells.

A-H, Related to Fig. 4C and 4D. Whole Gel image for Western blots using antibodies against phosphorylated-TAK1 (S412) (**A**), phosphorylated-p54 JNK and p46 JNK (T183/Y185) (**B**), phosphorylated-c-Jun (S63) (**C**), TAK1 (**D**), p54 JNK and p46 JNK (**E**), c-Jun (**F**), β-actin (**G**), and γH2AX (**H**).

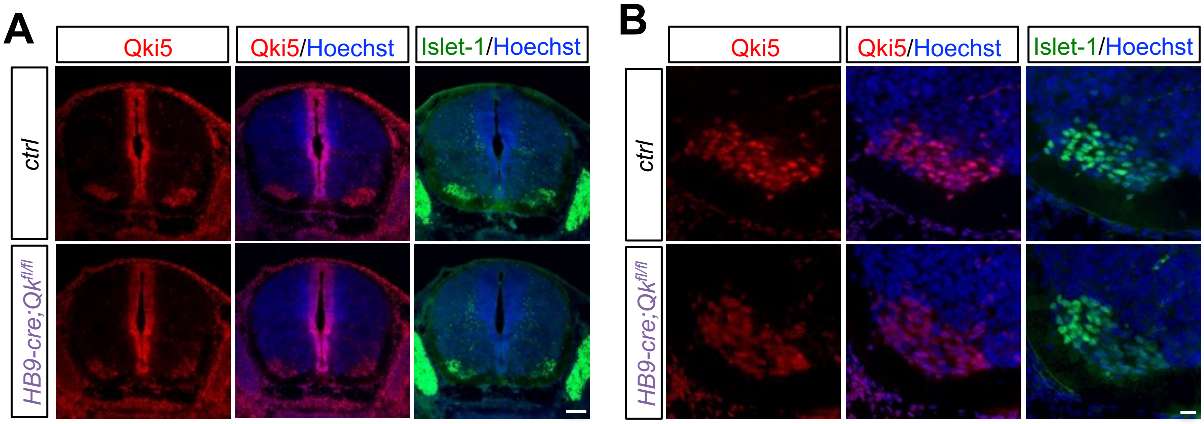

Fig. S10. Immunohistochemistry of *HB9-cre/+;Qkfl/fl* and *control* mouse spinal cords.

A, Double immunostaining using antibodies against Islet-1 (green) and Qki5 (red) in E13.5 *HB9-cre/+; Qkfl/fl* and *control* mouse spinal cords. Scale bar, 100 μm.

B, Enlarged view of the ventral horn in (**A**), indicating that Qki5 protein expression in Islet-1-positive MNs remained at this timepoint. Scale bar, 20 μm.

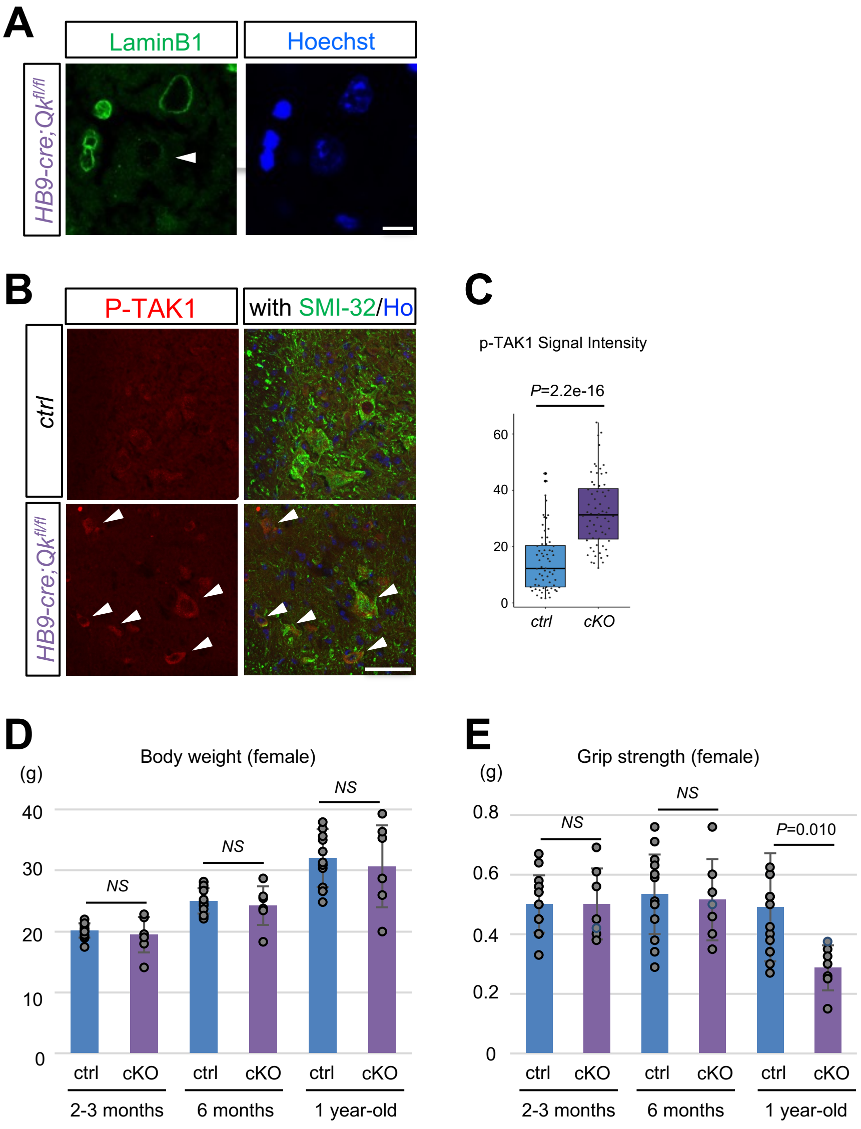

Fig. S11. phenotypes of *HB9-cre/+;Qkfl/fl* and control mice.

A, Another representative image of immunostaining for lamin-B1 (green) related to Fig. 5D. Qki5-deficient motor neurons show disruption of the nuclear membrane structure. An arrowhead indicates the missing of the lamin-B1 positive nuclear membrane. Scale bar, 10 μm.

B, Double immunostaining using antibodies against Phosho-TAK1 (red) and SMI-32 (green) in *HB9-cre/+; Qkfl/fl* and *control* mouse spinal cords at 1 month of age. Scale bar, 50 μm.

C, Box and whisker plot showing the increase of Phospho-TAK1 signal intensity in *HB9-cre/+; Qkfl/fl* spinal cord. Box represent the mean ± SD of three independent experiments. Dots represent for the signal intensity; N=64 MNs for each genotype-Welch's Two sample *t*-test.

D, Bar graphs indicate the average body weight (g) of female mice (right, n=7 for cKO mice and n=15 for littermate controls) at 2-3 months, 6 months old, and 1 year old of age. Error bars show the S.D., assessed with a two-tailed Student's *t*-test.

E, Bar graphs showing the grip strength test of female mice shown in D. Error bars show the S.D., assessed with a two-tailed Student's *t*-test.

Table S1. Gene list with transcript level changes between *siNC* and *siQk* in NSC-34 cells

rank	Dene ID	Gene Name	logFC	logCPM	P-Value	FDR
1	19317	Qk	2.960	5.930	8.30718E-26	1.0523E-21
2	76441	Daam2	1.404	4.663	1.98999E-07	0.00126036
3	67217	L3hypdh	1.736	2.605	4.18281E-07	0.00176612
4	20619	Snap23	1.201	4.842	3.0594E-05	0.09688363

Table S2. Gene list with transcript level changes between *siNC* and *siQKI* in hiPSC-MNs

1 of 2

rank	gene.ids	gene.names	logFC	logCPM	PValue	p.adj
1	8082	SSPN	1.37699787	2.81857008	1.24E-20	1.80E-16
2	9444	QKI	1.60864773	7.13171693	2.21E-18	1.61E-14
3	494470	RNF165	0.61143165	4.89582802	7.73E-11	3.74E-07
4	7168	TPM1	0.44968231	8.37978121	4.54E-09	1.65E-05
5	23428	SLC7A8	0.58670312	3.85409277	1.63E-07	0.00047318
6	57214	KIAA1199	0.63148606	4.17078406	2.03E-07	0.0004769
7	154664	ABCA13	1.01151172	1.0738035	2.46E-07	0.0004769
8	100500862	MIR3648	1.80991623	2.60920819	2.63E-07	0.0004769
9	162632	USP32P1	0.97732	0.58156294	2.07E-06	0.00334479
10	3426	CFI	0.45563996	6.47264316	2.51E-06	0.00364232
11	5806	PTX3	1.16436611	2.83890559	2.93E-06	0.00387207
12	10392	NOD1	0.90247224	1.54399019	3.82E-06	0.00431353
13	57481	KIAA1210	0.9379618	1.40797683	3.86E-06	0.00431353
14	387758	FIBIN	0.54005056	3.83080564	4.66E-06	0.00483597
15	58528	RRAGD	-0.4273717	5.73501576	6.29E-06	0.00608906
16	121506	ERP27	-1.0028049	0.58526422	7.37E-06	0.00668874
17	5764	PTN	0.49588091	5.73929117	8.06E-06	0.0068851
18	1360	CPB1	1.2925683	0.318027	9.36E-06	0.00755501
19	8728	ADAM19	-0.3230545	8.58845251	1.08E-05	0.00818122
20	27031	NPHP3	0.60337136	5.19968836	1.13E-05	0.00818122
21	100507246	LOC100507246	-0.3541031	6.76842486	1.20E-05	0.00828594
22	84376	HOOK3	0.31401233	7.41500889	1.32E-05	0.00873933
23	8564	KMO	0.80205635	1.76620292	1.75E-05	0.01104969
24	81035	COLEC12	0.46418231	6.26688508	2.49E-05	0.01510206
25	222256	CDHR3	0.652004	2.19001694	2.74E-05	0.01589401
26	9037	SEMA5A	0.34541894	7.0606892	3.15E-05	0.01758385
27	51071	DERA	0.65289748	2.63662198	3.44E-05	0.01848605
28	54930	HAUS4	-0.7788794	2.02558034	4.19E-05	0.0210852
29	727	C5	0.66562302	2.95806709	4.21E-05	0.0210852
30	55765	C1orf106	0.58708552	2.62476791	5.00E-05	0.02421902
31	51302	CYP39A1	0.88444869	0.65873268	5.39E-05	0.02524956
32	10493	VAT1	-0.6713458	7.82859533	5.66E-05	0.02567643
33	10581	IFITM2	0.66494493	2.73291154	5.92E-05	0.026058
34	7474	WNT5A	-0.4476402	4.67047025	6.34E-05	0.02644094
35	134957	STXBP5	-0.3206079	6.35477516	6.37E-05	0.02644094
36	2918	GRM8	0.68571074	1.88064262	7.00E-05	0.02650194
37	100507436	MICA	-0.9443839	0.93560477	7.10E-05	0.02650194
38	8854	ALDH1A2	0.37422323	5.14570884	7.11E-05	0.02650194
39	10471	PFDN6	-0.8928106	1.02142961	7.11E-05	0.02650194
40	171024	SYNPO2	0.57505062	3.89456353	7.70E-05	0.02744344

rank	gene.ids	gene.names	logFC	logCPM	PValue	p.adj
41	55723	ASF1B	0.91939438	0.91270219	7.74E-05	0.02744344
42	4893	NRAS	-0.3609699	6.90579439	8.28E-05	0.02837493
43	3218	HOXB8	0.3411293	6.80686708	8.40E-05	0.02837493
44	7545	ZIC1	0.37514942	5.39961838	9.20E-05	0.02996587
45	93556	EGFEM1P	0.40956784	5.51729954	9.28E-05	0.02996587
46	51573	GDE1	-0.3168603	6.19607652	9.57E-05	0.03023197
47	84971	ATG4D	-0.5159265	4.3661085	9.97E-05	0.03045341
48	26523	EIF2C1	-0.3292552	7.3208558	0.00010062	0.03045341
49	4501	MT1X	-1.1135948	0.7797315	0.00012138	0.03598921
50	283298	OLFML1	0.38616696	5.2695231	0.00014182	0.03948903
51	3635	INPP5D	-1.3594264	1.07590539	0.00014319	0.03948903
52	100289495	LOC100289495	-0.7484619	1.15597222	0.0001467	0.03948903
53	6092	ROBO2	0.29718653	7.2972451	0.00014677	0.03948903
54	11096	ADAMTS5	0.55615914	3.71025424	0.00014678	0.03948903
55	54715	RBFOX1	0.34243032	6.00820652	0.0001498	0.03956846
56	6335	SCN9A	0.38489417	6.53004692	0.00017464	0.04530618
57	50650	ARHGEF3	-0.5209032	4.7787264	0.00018253	0.04652394
58	1435	CSF1	-0.4955864	4.26316934	0.00019532	0.04892393
59	11280	SCN11A	0.58523076	2.28799645	0.00021212	0.05223119
60	6474	SHOX2	-0.7914854	1.27043164	0.00022174	0.05294495
61	130576	LYPD6B	0.80525385	0.62451771	0.00022302	0.05294495
62	793	CALB1	-0.6510365	3.49309402	0.00022595	0.05294495
63	6047	RNF4	-0.3337882	6.45120206	0.00023916	0.05515211
64	4781	NFIB	0.3860038	4.98193442	0.00028057	0.0636901
65	3222	HOXC5	0.4056181	4.47430373	0.00029427	0.06520973
66	2567	GABRG3	0.67755074	2.66573687	0.00029624	0.06520973
67	794	CALB2	-0.908077	2.63154938	0.00031592	0.06850327
68	3780	KCNN1	0.36879854	4.47248064	0.00032654	0.0688456
69	55266	TMEM19	-0.4008024	4.42149033	0.00032794	0.0688456
70	340542	BEX5	0.65900059	1.61604209	0.00033172	0.0688456
71	661	POLR3D	-0.4571389	4.91675012	0.00037114	0.07566718
72	284	ANGPT1	0.51966039	4.46258989	0.000375	0.07566718
73	57335	ZNF286A	-0.2840039	6.76402425	0.00039204	0.07802036
74	4685	NCAM2	0.30468473	5.8838909	0.00040291	0.07910075
75	85445	CNTNAP4	0.5842403	2.92372419	0.00041272	0.07983906
76	283234	CCDC88B	0.66493736	1.20501714	0.00041766	0.07983906
77	143686	SESN3	-0.3433581	6.63752319	0.00042572	0.08032315
78	389692	MAFA	0.38577325	5.22068174	0.00043722	0.0814349
79	23596	OPN3	0.56524855	2.56657011	0.00045617	0.08388904
80	144501	KRT80	0.94341392	0.13391053	0.00048105	0.08735793
81	64093	SMOC1	0.9770788	0.31521387	0.00050599	0.08998466
82	4880	NPPC	-0.8860453	0.90516513	0.00050839	0.08998466
83	32	ACACB	0.53202345	2.78785826	0.00051409	0.08998466
84	283377	SPRYD4	-0.5157049	2.6034352	0.00052837	0.09138283
85	1018	CDK3	-0.6694751	2.05050101	0.00056837	0.09702146
86	116151	FAM210B	-0.3742472	5.14040138	0.00057433	0.09702146
87	827	CAPN6	0.69890364	1.15805563	0.00058513	0.0977092

Table S3. Qki5 regulates multiple types of alternative splicing pattern in NSC-34 cells

AS type	# of events	% of events	Enrichment	p-value	Image
CE	389	64.0	1.5	2.20E-16	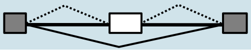
TACE	78	12.8	1.2	0.085	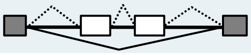
MXE	27	4.4	1.4	0.106	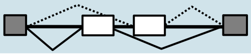
A5SS	28	4.6	0.5	1.96E-06	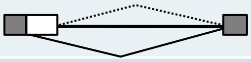
A3SS	38	6.3	0.2	3.94E-04	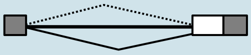
IR	48	7.9	0.9	0.471	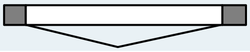

(CE) Cassette exon; (TACE) tandem cassette exon; (MXE) mutually exclusive exon; (A5SS) alternative 5′ site; (A3SS) alternative 3′ site; (IR) intron retention.
N=608, cut-off |DI|>0.1, p<0.01, and FDR<0.1. *p*-value are calculated by Fisher's exact test.

Table S4. Qki5 regulates multiple types of alternative splicing pattern in hiPSC-MNs

AS type	# of events	% of events	Enrichment	p-value	Image
CE	246	57.1	1.4	2.79E-10	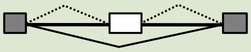
TACE	59	13.7	0.8	0.027	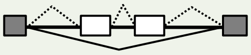
MXE	19	4.4	0.7	0.078	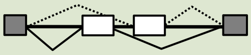
A5SS	14	3.2	0.4	1.04E-04	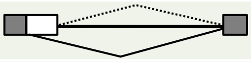
A3SS	58	13.5	0.7	3.00E-04	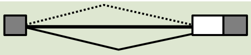
IR	35	8.1	1.4	0.036	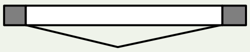

(CE) Cassette exon; (TACE) tandem cassette exon; (MXE) mutually exclusive exon; (A5SS) alternative 5′ site; (A3SS) alternative 3′ site; (IR) intron retention.
N=431, cut-off |DI|>0.1, p<0.01, and FDR<0.1. *p*-value are calculated by Fisher's exact test.

Table S5. Metascape enrichment pathway analysis using a list of genes with AS changes between *siNC* and *siQk* in NSC-34 cells.

rank	Category	Term	Description	LogP	Log(q-value)	InTerm InList	Symbols
1	WikiPathways	WP310	mRNA processing	-14.747361	-10.4803539	51/453	Hnrnpd,Clk1,Clk2,Clk4,Ddx5,Eif4a2,Fxr1,Hnrnpk,Elavl2,Npm1,Rbmxl1,Rps24,Supt5,Synj2,Tial1,Zfp346,Lsm2,Exosc9,Hnrnpdl,Srrm1,Hnrnpa2b1,Prpf40a,Ptbp2,Ggcx,Adar,Srp19,Rbm25,Ttc14,Prpf18,Cpeb4,Fdx2,Dnajc8,Bclaf1,Dis3,Poldip3,Rbm26,Rbm12,Hnrnpm,Pum1,Pum2,Rbfox2,Clk3,U2af1,Rbm39,Rsrc2,Ptbp3,Eif4g3,Elmod3,Rbm10,Rbm41,Sbno1,Prpf19,Ccnl1,Mbnl2,Nup98,Hnrnph3,App,Atf2,Hsf1,Rbbp6,Tbp,Exosc10,Eef1d,Fip1l1,Luc7l,Paxbp1,Aar2,Samd4,Pym1,Dbr1,Pan2,Cbll1,Ythdf2,Smg7,Prpf39,Vegfa,Angel2,Gtf2h1,Psmd2,Gnl3,Rpl4,Rpl22l1,Gtf2f2,Apobec3,Csnk1d,Eif4g1,Rpl23a,Mettl5,Trmt5,Rpusd1,Spout1
2	GO Biological Processes	GO:0051640	organelle localization	-13.443058	-9.477080862	53/522	Dctn1,Incenp,Kif13a,Kif1b,Kifc5b,Mef2a,Map6,Mapt,Myo1b,Myo5a,Npm1,Snap91,Snap23,Stk11,Synj2,Tcf7l2,Terf1,Bves,Tcirg1,Bin1,Esyt2,Exoc7,Stx5a,Tacc2,Syne1,Exoc2,Trappc13,Trappc6a,Dock7,Chmp2a,Exoc1,Spag9,Pinx1,Opa1,Bicdl1,Clasp2,Clasp1,Ppfia3,Bicd2,Eml4,Kat5,Ndel1,Sec16b,Pard3,Akap9,Synj1,Rims2,Cdk5rap2,Cdc42bpa,Sec16a,Spout1,Stx16,Picalm
3	GO Biological Processes	GO:0031344	regulation of cell projection organization	-10.874606	-7.120667241	61/761	Agrn,App,Cask,Cit,Dync1i2,Dnm2,Dscam,Evl,Ezh2,Fgfr1,Fxr1,Grin1,Hnrnpk,Igf1r,Itga6,Lrp8,Ltk,Mns1,Map6,Mapt,Neo1,Nin,Numb,Ptprf,Ptprs,Dennd5a,Sema3f,Cyfip1,Slit2,Snap25,Snap91,Stk11,Vegfa,Vldlr,Nckap1,Camk1,Golga4,Sh3glb1,Ift20,Syne1,Dynlt2b,Rpl4,Retreg3,Entr1,Ppp1r35,Cdhr5,Grip1,Opa1,Cyld,Ndel1,Obsl1,Braf,Rap1gap,Picalm,Cdk10,Sarm1,Nrcam,Islr2,Abi2,Dixdc1,Tnik,Nf2,Tcf7l2,Tenm4,Adgre5,Prpf19,Bin1,Nap1l1,Hes6,Clstn1,Dock7,Mtmr2,Pard3,Adgrl2,Synj1,Ythdf2,Wasf3,Bcl2l11,Cacna1c,Epb41l3,Gnas,Hsf1,Idh3g,Npm1,Ptch1,Ncor1,Atxn2,Smarca4,Tead1,Morf4l1,Nubp1,Acsl4,Exosc9,Htra2,Sgip1,Mrgbp,Usp47,Cep43,Clasp2,Sbf1,Kat5,Rims2,Eif4g1,Ankrd26,Add1,Npr2,Clcn3,Sptan1,Tpm1,Add3,Inf2,Cyrib
4	GO Biological Processes	GO:0010638	positive regulation of organelle organization	-10.462677	-6.894639142	48/529	Hnrnpd,Bcl2l11,Dctn1,Asap1,Evl,Irgm1,Igf1r,Cd47,Mns1,Mapt,Nf2,Nin,Sh2b1,Cyfip1,Tcp1,Terf1,Tpm1,Macroh2a1,Add3,Gnl3,Bin1,Nckap1,Hnrnpa2b1,Sh3glb1,Htra2,Fis1,Resf1,Entr1,Ppp1r35,Opa1,Mff,Ckap5,Kat5,Akap9,Synj1,Slain1,Braf,Ddhd1,Cdk5rap2,Git1,Cdc27,Pphln1,Slf2,Ambra1,Eif4g3,Wasf3,Abi2,Acd,Agrn,Dst,Mpdz,Map6,Npm1,Stk11,Nubp1,Fbxw5,Stmn4,Ccnl1,Tacc2,Dynlt2b,Chmp2a,Camsap3,Ttc21b,Cyld,Clasp2,Clasp1,Bicd2,Eml4,Phldb1,Ccdc40,Dixdc1,Add1,Ect2,Gpm6b,Slit2,Sptan1,Afap1,Arhgap17,Cyrib,Ppfia1,Cdk10,Dnm2,Flot1,Hsf1,Snap91,Vegfa,Adgre5,Map4k4,Clstn1,Ndel1,Adgrl2,Eif4g1,App
5	WikiPathways	WP1983	Splicing factor NOVA regulated synaptic proteins	-9.5993469	-6.177437548	13/42	Agrn,Ank3,Cask,Epb41l1,Epb41l2,Epb41l3,Grin1,Neo1,Plcb4,Cadm1,Clstn1,Clasp1,Rap1gap
6	GO Biological Processes	GO:0032990	cell part morphogenesis	-9.3791479	-6.06248848	47/550	Abi1,Ank3,App,Dnm2,Dscam,Dst,Enah,Epb41l3,Evl,Flot1,Igf1r,Lrp8,Mef2a,Map6,Mapt,Myo9b,Neo1,Nfib,Nin,Numb,Ptch1,Atxn2,Sema3f,Cyfip1,Slit2,Snap91,Stk11,Vegfa,Vldlr,Tenm2,Map4k4,Nckap1,Fis1,Dock7,Nexn,Prmt3,Grip1,Opa1,Mff,Ndel1,Rbfox2,Unc5b,Trio,Picalm,Kif20b,Nrcam,Tnik,Agfg1,Tpm1,Tenm4,Ckap5,Mtmr2,Cit,Fgfr1,Gpm6b,Mpdz,Ptprf,Acsl4,Ift20,Stmn4,Camsap3,Fryl,Bicdl1,Git1,Abi2,Add1,Ect2,Fat1,Smarca4,Bves,Cadm1,Pard3,Flnb
7	Reactome Gene Sets	R-MMU-199991	Membrane Trafficking	-9.3294958	-6.06248848	46/533	App,Dctn1,Dync1i2,Dnm2,Epn2,Eps15,Eps15l1,Fnbp1,Agfg1,Kif1b,Kifc5b,Dennd5a,Reps1,Snap91,Snap23,Sort1,Synj2,Tfg,Tpd52,Bin1,Golga4,Stx5a,Necap2,Trappc13,Trappc6a,Dctn4,Chmp2a,Sgip1,Clvs1,Arfrp1,Bicd2,Sbf1,Pum1,Sec16b,Csnk1d,Dennd2a,Lman2l,Sec24c,Vps52,Sec16a,Stx16,Arfgap1,Picalm,Kif20b,Sbf2,Mia3,Snap25,Atp6ap1,Snx12,Slc66a2,Entr1,Spag9,Grip1,Acap2,Erc1,Snx32,Laptm5
8	GO Biological Processes	GO:0000226	microtubule cytoskeleton organization	-8.4275783	-5.435028004	44/530	Bnip2,Dctn1,Dst,Incenp,Kifc5b,Mark3,Mns1,Mpdz,Map6,Mapt,Nin,Npm1,Ncor1,Tubd1,Stmn4,Tacc2,Cep20,Dock7,Camsap3,Tube1,Haus7,4933427D14Rik,Cep43,Ckap5,Clasp2,Clasp1,Bicd2,Eml4,Kat5,Ndel1,Pard3,Obsl1,Akap9,Lrrc49,Csnk1d,Slain1,Ccdc40,Armc2,Cdk5rap2,Chd3,Cdc42bpa,Haus6,Prc1,Ttll5,Dync1i2,Ywhag,Cenpu,Cenpi,Nup98,Cenpt,Asap1,Tcp1,Exoc7,Ift20,Dynlt2b,Exoc2,Exoc1,Ttc21b,Chmp2a,Abi1,Add1,App,Enah,Evl,Fat1,Agfg1,Mef2a,Mmp11,Myo1b,Myo5a,Sorbs1,Tpm1,Tpm3,Arhgap17,Abi2,Mia3,Cdc25c,Psmd2,Cdc27

rankZ	Category	Term	Description	LogP	Log(q-value)	InTerm InList	Symbols
9	GO Biological Processes	GO:0051668	localization within membrane	-8.3778642	-5.411886789	44/532	Adam22,Agrn,Ank3,Cacnb1,Cask,Dnm2,Epb41l3,Flot1,Flot2,Grin1,Kif13a,Myo5a,Nrxn2,Ptch1,Snap25,Snap23,Timm9,Golga4,Atp6ap1,Sh3glb1,Ift20,Snx12,Clstn1,Srp19,Exoc2,Entr1,Zdhhc3,Exoc1,Grip1,Zdhhc20,Clasp2,Arfrp1,Acap2,Slmap,Fam126a,Pard3,Braf,Rapgef6,Abhd17a,Git1,Vps52,Bag6,Sec16a,Tnik,Kif1b,Nubp1,Eps15,Laptm5,Myo1b,Sort1,Tfg,Atp9b,Stx5a,Trappc13,Trappc6a,Bicd2,Sec16b,Nrbp1,Lman2l,Sec24c,Mia2,Mia3,Myo18a
10	GO Biological Processes	GO:0018193	peptidyl-amino acid modification	-7.8832884	-5.02119542	55/778	Abi1,Atf2,Cit,Clk1,Clk2,Clk4,Ezh2,Fgfr1,Igf1r,Mark3,Msl3,Mast2,Nfya,Nmt2,P4ha2,Smarca4,Stk11,Morf4l1,Tlk2,Mapk12,Camk1,Ggcx,Mast1,Brd4,Asph,Fkbp11,Naa16,Rps6ka6,Eef1akmt1,Zdhhc3,Ndufab1,Prmt3,Hpf1,Setd5,Mrgbp,Mettl23,Cxxc1,Zdhhc20,Ehmt1,Kat5,Arid4b,Clk3,Csnk1d,Ehmt2,Brd1,Bag6,Cdc42bpa,Kansl3,Uspl1,Wnk1,Cdk10,Phf20l1,Ttll5,Mast4,Abi2,App,Cask,Gtf2h1,Hsf1,Incenp,Ltk,Prkab1,Snrk,Tbp,Map4k4,Strada,Braf,Erc1,Trio,Npr2,Map4k5,Tnik
11	Reactome Gene Sets	R-MMU-194315	Signaling by Rho GTPases	-7.4762252	-4.686339101	48/652	Abi1,Cdc25c,Cit,Dync1i2,Dst,Ect2,Evl,Sh3pxd2a,Flot1,Flot2,Fnbp1,Incenp,Ktn1,Nf2,Rbbp6,Cyfip1,Snap23,Ywhag,Git2,Add3,Nckap1,Srrm1,Atp6ap1,Stx5a,Ralgapa1,Tpm3,Erbin,Fam135a,Arhgap17,Cenpu,Acbd5,Ckap5,Clasp2,Clasp1,Ndel1,Gopc,Cenpi,Arhgef11,Git1,Trio,Cdc42bpa,Prc1,Picalm,Dock4,Wasf3,Nup98,Cenpt,Abi2
12	GO Biological Processes	GO:0051301	cell division	-7.1544157	-4.392558351	40/505	Ank3,Cdc25c,Cit,Clta,Dctn1,Ect2,Epb41l2,Fgfr1,Incenp,Kif13a,Numb,Septin5,Terf1,Tial1,Top2a,Jtb,Gnl3,Fubp1,Exoc7,Septin6,Fign,Exoc2,Dock7,Entr1,Chmp2a,Exoc1,Zfyve19,Haus7,Ckap5,Clasp2,Clasp1,Eml4,Pard3,Ythdf2,Cdc27,Spout1,Prc1,Kif20b,Cenpt,Dixdc1,Atf2,Bcl2l11,Bnip2,Ezh2,Hsf1,Kifc5b,Nin,Npm1,Ncor1,Stk11,E2f6,Nasp,Brd4,Tacc2,Usp29,Strada,Pinx1,4933427D14Rik,Cacul1,Kat5,Ndel1,Csnk1d,Ehmt2,Cdk5rap2,Chd3,Bag6,Haus6,Tubd1,Tube1
13	GO Biological Processes	GO:0051642	centrosome localization	-6.2932433	-3.65970438	9/33	Nin,Nubp1,Ift20,Syne1,Bicd2,Ndel1,Pard3,Akap9,Spout1
14	GO Biological Processes	GO:0034453	microtubule anchoring	-6.0981304	-3.493880856	8/26	Dctn1,Nin,Cep20,Camsap3,Cep43,Clasp2,Clasp1,Bicd2
15	GO Biological Processes	GO:0007163	establishment or maintenance of cell polarity	-5.6359216	-3.121940788	21/208	Dctn1,Dst,Fat1,Igf1r,Myo9b,Snap91,Stk11,Tcirg1,Nckap1,Ift20,Dock7,Camsap3,Ckap5,Clasp2,Clasp1,Kat5,Ndel1,Pard3,Frmd4a,Cdk5rap2,Myo18a
16	GO Biological Processes	GO:1903829	positive regulation of protein localization	-5.6237823	-3.12020294	36/495	Ank3,App,Cask,Ect2,Epb41l2,Hnrnpk,Mapt,Myo5a,Npm1,Sorbs1,Stk11,Tcf7l2,Tcp1,Vegfa,Gnl3,Acsl4,Camk1,Sh3glb1,Xpo4,Asph,Fis1,Entr1,Exoc1,Pinx1,Cyld,Mff,Clasp2,Hnrnpm,Glis2,Frmd4a,Abhd17a,Atp2c1,Kif20b,Abat,Zfp384,Myo18a,Rhbdf1,Neo1,Snap25,Snap91,Terf1,Tcirg1,Snx12,Tbc1d1,Midn,Ndel1,Gopc,Hmgn3,Os9,Rap1gds1,Picalm,Bves,Dynlt2b,Arv1,Ttc21b,Mtmr2
17	GO Biological Processes	GO:0060627	regulation of vesicle-mediated transport	-5.6107538	-3.114598416	42/623	Add1,App,Cask,Dnm2,Epn2,Flot1,Hnrnpk,Cd47,Lgals9,Myo5a,Numb,Septin5,Atxn2,Snap91,Snap23,Vegfa,Bves,Git2,Cadps,Bin1,Sh3glb1,Snx12,Exoc2,Letmd1,Chmp2a,Sgip1,Ckap5,Clasp2,Clasp1,Mtmr2,Pard3,Gopc,Synj1,Cbll1,Braf,Rap1gap,Rims2,Git1,Arfgap1,Picalm,Atp2c1,Myo18a
18	GO Biological Processes	GO:0010564	regulation of cell cycle process	-5.3509829	-2.890155526	46/727	App,Atf2,Cdc25c,Cit,Dctn1,Ect2,Ezh2,Fgfr1,Hsf1,Igf1r,Incenp,Kif13a,Npm1,Sh2b1,Smarca4,Smarcb1,Nubp1,Macroh2a1,Prpf19,Fbxw5,Bin1,Sh3glb1,Prpf40a,Ccnl1,Brd4,Pbrm1,Smarcc2,Entr1,Chmp2a,Ppp1r35,Zfyve19,Pinx1,Usp47,Kat5,Obsl1,Eif4g1,Ythdf2,Cdk5rap2,Git1,Cdc27,Slf2,Ambra1,Eif4g3,Prc1,Cdk10,Kif20b,Bcl2l11,Ccpg1,Ptch1,Angel2,Cyld
19	GO Biological Processes	GO:0033044	regulation of chromosome organization	-5.1850833	-2.798889559	22/240	Hnrnpd,Cit,Incenp,Smarca4,Smarcb1,Tcp1,Terf1,Top2a,Macroh2a1,Gnl3,Exosc10,Hnrnpa2b1,Pbrm1,Resf1,Smarcc2,Pinx1,Kat5,Cdk5rap2,Cdc27,Pphln1,Slf2,Acd,Telo2,Npm1,Sh2b1,Vegfa
20	GO Biological Processes	GO:0050807	regulation of synapse organization	-5.0536951	-2.689777678	23/263	Agrn,App,Cask,Dctn1,Grin1,Hnrnpk,Lrp8,Ptprf,Ptprs,Sema3f,Cyfip1,Snap91,Adgre5,Camk1,Clstn1,Setd5,Opa1,Adgrl2,Eif4g1,Abhd17a,Picalm,Nrcam,Abi2,Asap1,Pbrm1

Table S6. Metascape enrichment pathway analysis using a list of genes with AS changes between *siNC* and *siQKI* in hiPSC-MNs.

1 of 2

rank	Category	Term	Description	LogP	Log(q-value)	InTerm_InList	Symbols
1	GO Biological Processes	GO:0030029	actin filament-based process	-23.163	-18.801	56/796	ACTN1,ADD3,ANK2,RHOC,CACNA1C,CACNA1D,CACNA2D1,CNN2,EPB41L2,FAT1,FLNB,GAB1,ILK,JAK2,KCND3,ATXN3,NF1,NF2,PFN2,SPTAN1,SVIL,TJP1,TPM1,TPM2,TPM3,SORBS2,SLIT2,PICK1,PHACTR2,ABI1,ACTR2,AKAP9,ABI2,BAIAP2,SORBS1,NCKAP1,FERMT2,AKAP13,AKAP11,LIMCH1,CLASP2,MPRIP,CLASP1,PIP5K1C,ARFIP1,MINK1,SHANK1,EVL,LIMA1,CYRIB,CCDC88A,MICAL3,MLST8,FMNL3,CARMIL2,MYO18A,DST,COL5A1,GOLGA2,NUMA1,SPAST,PXDN,USP9X,NIN,NDEL1,PLXNB1,PHLDB1,BICD2,AFAP1,DNM1L,ASIC2,DNM2,EIF4G1,NRXN1,MAP4K4,CROCC,CLSTN1,ADGRL2,FN1,NRCAM,SEMA3F,CNTN2,PICALM,IST1,STXBP1,CARD8,RPL13A,STXBP5,ATP2B1,ZNF205,MACROH2A1,CAMKK2,PHIP,ACD
2	GO Biological Processes	GO:0032989	cellular component morphogenesis	-20.601	-16.716	51/743	ANK2,ANK3,DNM2,FN1,ILK,LAMA2,MCF2,NRCAM,PLXNB1,PPP3CA,ROBO1,ROBO2,SEMA3F,SPAST,STXBP1,CNTN2,TPM1,VLDLR,LRP8,USP9X,PICALM,NUMB,SLIT2,NRXN3,NRXN1,MAP4K4,RAPGEF2,RIMS2,IST1,ABI1,DNM1L,PQBP1,ACTR2,ABI2,BAIAP2,POSTN,NCKAP1,AKAP13,NFASC,CLASP2,PHLDB1,CLASP1,RBFOX2,MINK1,SHANK1,NIN,PARD3,CAPRIN2,NDEL1,DOCK7,PIKFYVE,ACTN1,FAT1,FLNB,FERMT2,SPRY4,ASIC2,CTSC,MBD1,NF1,NF2,CLSTN1,ADGRL2,NPHP3,NSUN5,CAMK2G,DGUOK,ITGA6,PFN2,SNAP25,CROCC,SDCCAG8,CRTC1,SGSM3,EVL,CCDC88A,CARMIL2,TNC,JAK2,PPP1R12A,HDAC9,PRMT2,SORBS2,EIF4G1,FHL1,CCAR2,KIAA1109,SMAD2,CELF1,NLGN4X,PLEKHA1,CTC1
3	GO Biological Processes	GO:0034330	cell junction organization	-20.281	-16.521	49/695	ASIC2,ACTN1,ANK2,ANK3,RHOC,DST,CAST,EIF4G1,FN1,TNC,ITGA6,AFDN,NF2,NRCAM,PLXNB1,PTPRK,ROBO2,SEMA3F,CNTN2,TJP1,YWHAZ,LRP8,PKP4,NUMB,NRXN1,NRXN2,MAP4K4,PICK1,RAPGEF2,DNM1L,ACTR2,ABI2,BAIAP2,SORBS1,FERMT2,CLSTN1,LIMCH1,NFASC,CLASP2,ADGRL2,ADGRL3,CLASP1,PIP5K1C,SHANK1,SDK2,PARD3,NLGN4X,CAPRIN2,LRFN5
4	Reactome Gene Sets	R-HSA-9716542	Signaling by Rho GTPases, Miro GTPases and RHOBTB3	-13.733	-10.626	41/723	ACTN1,ADD3,RHOC,DST,KTN1,MCF2,PPP1R12A,NF2,PFN2,PLXNB1,SPTAN1,TMPO,TPM3,YWHAZ,USP9X,PICALM,PKP4,ABI1,ACTR2,FAM13A,ABI2,BAIAP2,NCKAP1,FERMT2,AKAP13,CLASP2,ANKLE2,CLASP1,TAX1BP3,EVL,PHIP,CCDC88A,ERBIN,FAM135A,CENPT,NDEL1,DOCK7,RHOT2,FMNL3,EPSTI1,RALGAPA1
5	Reactome Gene Sets	R-HSA-9675108	Nervous system development	-12.610	-9.591	35/577	ANK2,ANK3,RHOC,CACNA1C,CACNA1D,COL5A1,COL6A3,DNM2,EIF4G1,FGFR1,GAB1,GRB10,GRIN1,LAMA2,NRCAM,PFN2,PLXNB1,ROBO1,ROBO2,RPS24,SCN3A,SPTAN1,CNTN2,TEAD1,UTRN,VLDLR,NUMB,SLIT2,ACTR2,NFASC,CLASP2,CLASP1,PIP5K1C,RPL13A,EVL
6	GO Biological Processes	GO:0051668	localization within membrane	-10.475	-7.645	34/651	ANK2,ANK3,ATP1B3,CHM,DNM2,AFDN,SNAP25,SRP9,STXBP1,SLMAP,PICALM,NUMB,NRXN1,NRXN2,PICK1,RAPGEF2,RAB11FIP3,SEC16A,EMC8,SORBS1,FERMT2,CLSTN1,NFASC,CLASP2,EXOC6B,PACS2,ADAM22,DENND4C,CCDC88A,EXOC1,PARD3,KIF13A,KIAA1109,PIKFYVE,EPB41L2,NUMA1
7	Reactome Gene Sets	R-HSA-6794361	Neurexins and neuroligins	-9.712	-6.958	11/55	APBA2,EPB41L2,GRIN1,STXBP1,CASK,NRXN3,NRXN1,NRXN2,DLGAP4,SHANK1,NLGN4X,CAMK2G,TJP1,ADCY9,CACNA2D1,KCNC3,KCND3,SNAP25,PICK1,CAMKK2,ANK3,SPAST,PICALM,ANKLE2,NF1,CNTN2,ACTR2,VLDLR,CRTC1,NSUN5,ITGA6,NRCAM,NFASC,CADM1,PBX3,ADAM22,EIF4G1,NAV2
8	GO Biological Processes	GO:0048813	dendrite morphogenesis	-9.707	-6.958	16/146	ILK,PPP3CA,VLDLR,LRP8,PICALM,RAPGEF2,ABI1,DNM1L,PQBP1,ACTR2,ABI2,BAIAP2,RBFOX2,MINK1,SHANK1,CAPRIN2,ACSL4,MCF2,CRTC1,RHOC,DNM2,FN1,PLXNB1,CNTN2,TPM1,RIMS2,POSTN,FERMT2,PHIP,PRPF40A,SPRY4,FMNL3,ACTN1,SEMA3F,NRXN1,NRXN2,PICK1,NLGN4X,ITGA6,NDEL1

rank	Category	Term	Description	LogP	Log(q-value)	InTerm_InList	Symbols
9	WikiPathways	WP4148	Splicing factor NOVA regulated synaptic proteins	-9.683	-6.944	10/42	ANK3,ATP2B1,CAMK2G,EPB41L2,GRIN1,PLCB4,CASK,CLSTN1,CLASP1,CADM1
10	GO Biological Processes	GO:0051017	actin filament bundle assembly	-9.153	-6.445	16/159	ACTN1,RHOC,NF2,PFN2,TJP1,TPM1,BAIAP2,SORBS1,FERMT2,LIMCH1,CLASP2,CLASP1,SHANK1,EVL,LIMA1,CCDC88A,AKAP13,CNN2,EPB41L2,MYO18A,KTN1,PPP1R12A,YWHAZ,ABI1,ACTR2,ABI2,NCKAP1,TAX1BP3,CENPT,NDEL1,FMNL3,ROBO1,SLIT2,ILK,AFDN,NF1,PTPRK,ROBO2,SEMA3F,EIF4G1,RPS24,RPL13A,FGFR1,FN1,MAP4K4,HDAC9,CAPN7
11	GO Biological Processes	GO:0030155	regulation of cell adhesion	-8.853	-6.165	34/751	ANK3,CBLB,DNM2,FN1,TNC,ILK,ITGA6,JAK2,LAMA2,ATXN3,AFDN,PPP1R12A,NF1,NF2,PLXNB1,PPP3CA,TJP1,TPM1,UTRN,PKP4,CASK,MAP4K4,POSTN,FERMT2,KIFAP3,LIMCH1,CLASP2,CLASP1,MINK1,CYRIB,ADAM22,WNK1,SPRY4,RC3H1,ACTN1,PTPRK,SORBS1,DST
12	GO Biological Processes	GO:0007268	chemical synaptic transmission	-8.738	-6.092	33/720	ASIC2,AMPH,APBA2,GRIN1,JAK2,LAMA2,ATXN3,PLCB4,PPP3CA,PTPRN2,SNAP25,STXBP1,CNTN2,LRP8,CASK,CADPS,NRXN1,NRXN2,PICK1,RAPGEF2,RIMS2,DNM1L,AKAP9,BAIAP2,DLGAP4,CLSTN1,KIF1B,CRTC1,PIP5K1C,MINK1,SHANK1,NLGN4X,STXBP5,ANK2,ANK3,ATP1B3,CACNA1C,CACNA1D,CACNA2D1,FHL1,KCND3,SCN3A,SLMAP,USP53,ATP2B1,CAMK2G,CTSC,PBX3,TPM1,PARD3,WNK1
13	GO Biological Processes	GO:0043087	regulation of GTPase activity	-8.421	-5.807	22/349	CBLB,ITGA6,AFDN,NF1,PLXNB1,PTPRN2,SBF1,PICALM,PKP4,MAP4K4,RAPGEF2,FERMT2,GAPVD1,SGSM3,TAX1BP3,SIPA1L2,MLST8,WNK1,NDEL1,DOCK7,MTSS2,RALGAPA1,RHOC,ATP1B3,FGFR1,FN1,GRIN1,JAK2,PPP1R12A,PFN2,ROBO1,TPM1,CARD8
14	GO Biological Processes	GO:0000226	microtubule cytoskeleton organization	-8.396	-5.798	30/628	DST,CHD3,GOLGA2,MARK3,ATXN3,PPP1R12A,NUMA1,PKD2,SPAST,CNTN2,TTLL4,CROCC,AKAP9,SDCCAG8,TTLL5,CUL9,CLASP2,PHLDB1,BICD2,CLASP1,SUN1,NIN,CCSER2,MAP7D1,CCDC88A,PARD3,SPAG16,NDEL1,DOCK7,DCLK2
15	GO Biological Processes	GO:0010256	endomembrane system organization	-8.088	-5.525	27/536	ANK2,ANK3,COL5A1,DNM2,GOLGA2,SNAP25,SPAST,STXBP1,YWHAZ,DYSF,IST1,SEC16A,DNM1L,AKAP9,EMC8,BAIAP2,SEC31A,CLASP2,ANKLE2,CLASP1,SYNE1,SUN1,MYOF,ESYT2,NDEL1,MTSS2,MYO18A
16	GO Biological Processes	GO:0007163	establishment or maintenance of cell polarity	-7.898	-5.356	17/221	RHOC,DST,FAT1,ILK,NUMA1,ACTR2,SDCCAG8,CLASP2,CLASP1,CYRIB,FRMD4A,ERBIN,PARD3,NDEL1,DOCK7,CARMIL2,MYO18A
17	GO Biological Processes	GO:0051129	negative regulation of cellular component organization	-7.778	-5.273	32/748	ADD3,DGUOK,DNM2,MCF2,PFN2,PPP3CA,ROBO2,SEMA3F,SPTAN1,STXBP1,SVIL,TJP1,PICALM,SLIT2,MAP4K4,PICK1,MACROH2A1,RAPGEF2,DNM1L,CARD8,CLASP2,CLASP1,RPL13A,SHANK1,EVL,LIMA1,CYRIB,PCID2,ACD,CTC1,ANKRD13B,CARMIL2,SPAST,MICAL3,SMARCC2,IST1,GOLGA2,CAMKK2,NDEL1
18	GO Biological Processes	GO:0007420	brain development	-7.710	-5.212	32/753	ATP2B1,BPTF,GRIN1,HSPG2,NF1,NF2,PBX3,PPP3CA,ROBO1,ROBO2,SMARCA1,CNTN2,LRP8,NUMB,SLIT2,RAPGEF2,SEC16A,BAIAP2,CELF1,ADGRL2,ADGRL3,SUN1,RBFOX2,TMX2,NIN,NSUN5,MEIS3,NLGN4X,METTL14,NDEL1,DOCK7,DCLK2,PLEKHA1
19	GO Biological Processes	GO:0051640	organelle localization	-7.593	-5.112	26/529	DNM2,NUMA1,SNAP25,SPAST,STXBP1,YWHAZ,PICALM,CROCC,SEC16A,DNM1L,ACTR2,AKAP9,KIFAP3,CLASP2,EXOC6B,PACS2,BICD2,CLASP1,SUN1,SYTL2,PARD3,ESYT2,KIF13A,NDEL1,DOCK7,RHOT2,ANK3,RAB11FIP3,CLSTN1,EXOC1,KIAA1109,GOLGA2,PICK1,SEC31A,MYO18A
20	GO Biological Processes	GO:0042060	wound healing	-7.554	-5.084	23/424	ACTN1,RHOC,DST,CNN2,COL5A1,FN1,TNC,ILK,JAK2,SMAD2,NF1,PPP3CA,STXBP1,TPM1,DYSF,CASK,POSTN,FERMT2,CLASP2,CLASP1,FGFR1OP2,MYOF,CARMIL2,BPTF,NDEL1,FGFR1,GAB1,ITGA6,SEMA3F,TJP1,NUMB,SLIT2,MAP4K4,RAPGEF2,HDAC9,DNM1L,CAPN7,WNK1,DOCK7

Table S7. SEEK analysis showing that co-expression correlations with *Qk* and the targets

Rank	Gene	Entrez ID	Coexpression Score	P-Value	Description
1	FEZ2	9637	2.371	<0.0001	fasciculation and elongation protein zeta 2 (zygin II)
2	***ZEB2**	9839	2.337	<0.0001	zinc finger E-box binding homeobox 2
3	***MAP4K4**	9448	2.296	0.0001	mitogen-activated protein kinase kinase kinase kinase 4
4	AP1S2	8905	2.262	<0.0001	adaptor-related protein complex 1, sigma 2 subunit
5	CFL2	1073	2.243	0.0001	cofilin 2 (muscle)
6	DENND5A	23258	2.211	0.0001	DENN/MADD domain containing 5A
7	***SERINC1**	57515	2.185	0.0195	serine incorporator 1
8	ZCCHC24	219654	2.102	0.0009	zinc finger, CCHC domain containing 24
9	ARL8B	55207	2.090	0.0009	ADP-ribosylation factor-like 8B
10	C20ORF194	25943	2.071	0.0006	chromosome 20 open reading frame 194
11	MAP1LC3B	81631	2.044	0.0025	microtubule-associated protein 1 light chain 3 beta
12	GTDC1	79712	2.032	<0.0001	glycosyltransferase-like domain containing 1
13	SPG20	23111	2.014	0.0001	spastic paraplegia 20 (Troyer syndrome)
14	ZYG11B	79699	2.005	0.0035	zyg-11 homolog B (C. elegans)
15	NXPE3	91775	1.998	<0.0001	neurexophilin and PC-esterase domain family, member 3
16	EPS15	2060	1.995	0.0072	epidermal growth factor receptor pathway substrate 15
17	***ZEB1**	6935	1.977	0.0016	zinc finger E-box binding homeobox 1
18	MAP4	4134	1.976	0.0009	microtubule-associated protein 4
19	***SPAG9**	9043	1.974	0.0035	sperm associated antigen 9
20	MEF2A	4205	1.968	0.0009	myocyte enhancer factor 2A

* Indicates Qki5 direct target gene.
